# Synergistic dual-phase network in rigid-flexible PU/LAGP composite electrolytes for fast-ion-conducting solid-state lithium batteries

**DOI:** 10.3389/fchem.2026.1908013

**Published:** 2026-10-29

**Authors:** Xingyu Tang, Siqi Wang, Yiming Gou, Lixin Ma, Hao Wu, Peng Cui

**Affiliations:** 1 School of Environmental Science and Engineering, Nanjing Tech University, Nanjing, China; 2 Nanjing Pinnacle New Energy Ltd., Nanjing, China; 3 Zhengde Polytechnic College, Nanjing, China; 4 Nanjing Shiyuan Chemical Technology Co., Nanjing, China; 5 Shengli Oilfield Shengli Oil Production Plant, Oil Production Management Zone 3, Injection and Production Station 308, Dongying, China; 6 School of Mathematical and Physical Sciences, Nanjing Tech University, Nanjing, China

**Keywords:** all-solid-state battery, ionic conductivity, polymer electrolyte, polyurethane, polyurethane-based electrolyte

## Abstract

**Introduction:**

The practical implementation of all-solid-state lithium batteries (ASSLBs) is severely hindered by the trade-off between ionic conductivity and mechanical robustness. Additionally, “ion-trapping” effects give rise to sluggish interfacial lithium-ion transport, further restricting battery performance. Developing composite solid electrolytes that simultaneously resolve these two bottlenecks remains a critical challenge.

**Methods:**

A rigid-flexible composite electrolyte (denoted PLAP) was fabricated, which combines prelithiated polyurethane (PU) polymer matrix with Li_1.5_Al_0.5_Ge_0.5_P_3_O_12_ (LAGP) inorganic fillers. A soft-hard segmented molecular structure was tailored for the electrolyte matrix. Spectroscopic characterizations and theoretical computations were employed to analyse interfacial lithium-ion binding energy before and after terminal-group prelithiation (−OH → −OLi). For benchmark comparison, the non-prelithiated −OH-terminated PU/LAGP electrolyte from our previous work with identical composition and test conditions was adopted. Electrochemical measurements including tensile test, ionic-conductivity evaluation, symmetric-cell cycling and LiFePO_4_|PLAP|Li full-cell galvanostatic cycling were carried out to assess mechanical property and electrochemical performance.

**Results:**

The as-prepared PLAP composite electrolyte delivers a tensile strength of 5.47 MPa and an elongation of 192.4 %, benefiting from its custom soft-hard segmented architecture. Terminal-group prelithiation reduces the interfacial Li^+^ binding energy from 1.01 eV to 0.55 eV, which releases trapped lithium ions. The PLAP electrolyte achieves a high room-temperature ionic conductivity of 3.8 × 10^-4^ S/cm. Li||Li symmetric cells realise stable cycling over 750 h under an ultra-high current density of 10 mA cm^-2^. The LiFePO_4_|PLAP|Li full cells exhibit outstanding cycling durability, retaining 95.7 %, 91 % and 87.8 % of initial capacity after 2000 cycles at 0.2 C, 2 C and 5 C, respectively.

**Discussion:**

The terminal-group prelithiation strategy effectively mitigates ion-trapping by lowering interfacial Li^+^ binding energy, which improves ion-transport behaviour while preserving favourable mechanical performance originating from the rigid-flexible segmented structure. This work demonstrates a feasible material-design route toward long-lifespan and fast-charging liquid-free all-solid-state lithium-metal batteries.

## 1 Introduction

With the rapid expansion of the portable electronic device and electric vehicle markets, the societal requirement for power storage units characterized by high energy capacity and enhanced security has intensified significantly (Shin et al., 2023). Conventional liquid lithium-ion batteries (LIBs) have approached their theoretical energy density limit; more critically, the organic liquid electrolytes inherent in such batteries pose a severe risk of thermal runaway, which has become a critical bottleneck hampering their further industrialization and application (Ai et al., 2024). Consequently, ASSLBs employing all-solid-state electrolytes (ASSEs) to replace liquid electrolytes are widely recognized as the most promising next-generation energy storage solution, holding great potential to fundamentally address the safety issues and significantly enhance the energy density of the entire system (Jin et al., 2025).

Among current ASSEs, two primary classes are recognized: inorganic ceramic electrolytes and solid polymer electrolytes (SPEs) (Glynos et al., 2020). As a typical representative of Na Super-Ionic Conductor (NASICON) type inorganic solid electrolytes, Li_1.5_Al_0.5_Ge_0.5_P_3_O_12_ (LAGP) has garnered extensive attention due to its high ionic conductivity (10^−4^ S/cm at 25 °C) and wide electrochemical stability window (exceeding 6 V) (DeWees and Wang, 2019). Notably, LAGP exhibits excellent air stability, significantly outperforming garnet-type solid electrolytes (Zhang et al., 2024); more importantly, unlike sulfide-based solid electrolytes, no toxic H_2_S gas is generated during the application of LAGP, endowing it with distinctive advantages in terms of safety performance (Wang et al., 2021). Compared with the isostructural Li_1.5_Al_0.5_Ti_1.5_P_3_O_12_ (LATP) system, the introduction of the Ge element effectively broadens the electrochemical window and improves the stability against lithium metal (DeWees and Wang, 2019). However, the inherent brittleness of LAGP ceramics, elevated grain boundary resistance, and sluggish “solid-solid” interfacial kinetics with electrodes have severely restricted its practical application (Jiang et al., 2018; Gao et al., 2023). In contrast, SPEs such as polyethylene oxide (PEO), polycarbonate (PC), and polyurethane (PU) possess excellent flexibility, processability, and good interfacial compatibility (Dai et al., 2024), which can effectively make up for the inherent deficiencies of inorganic solid electrolytes. Nevertheless, SPEs suffer from intrinsic limitations including low ionic conductivity (<10^−5^ S/cm at 25 °C) and low lithium ion transference number (TLi^+^ < 0.2) at room temperature (Ma et al., 2017; Zhang et al., 2022).

To address the fundamental constraints of monolithic electrolytes, organic-inorganic hybrid solid electrolytes have become a prominent research focus in recent years (Cheng et al., 2021). The incorporation of inorganic fillers into polymer matrices aims to synergistically integrate the high conductivity of ceramics and the flexibility of polymers. Recent studies have demonstrated that the interaction between inorganic ceramic fillers and polymer segments can significantly reshape the ion transport pathways (Huang et al., 2025). Liu et al. (2017) confirmed that the ionic conductivity of composite electrolytes prepared by incorporating Li_0.33_La_0.557_TiO_3_ (with a grain size of approximately 50 nm) into polyacrylonitrile (PAN) was significantly improved from 2.1 × 10^−7^ S/cm to 2.4 × 10^−4^ S/cm compared with unfilled electrolytes. Zhang et al. (2020) developed a novel ceramic filler Li_6.4_La_3_Zr_1.4_Ta_0.6_O_12_ (LLZTO) and verified that the uniform dispersion of ceramic fillers in the polymer matrix could alleviate space-charge effects, homogenize Li-ion flux.

Although the aforementioned methods for preparing electrolytes have greatly improved the ionic conductivity of electrolytes, they still face the challenge that the polymer matrix cannot simultaneously achieve high ionic conductivity and excellent mechanical properties. Insufficient mechanical properties will lead to electrolyte membrane damage during high-rate cycling of batteries, thereby triggering short-circuit risks (Liang et al., 2023). Therefore, the selection of polymer matrix is a key prerequisite for the preparation of high-performance organic-inorganic composite electrolytes (Liu et al., 2015). Polyurethane has garnered substantial interest from scientists owing to its distinctive “soft-hard” segment structure (Cui et al., 2024): the “soft segment” can effectively solvate lithium salts, homogeneously disperse active fillers, and facilitate functional group modification; meanwhile, the “hard segment” can provide good mechanical properties for polymer molecules. Despite the advantages of polyurethane-ceramic composite electrolytes, their practical deployment is severely limited by interfacial Li^+^ trapping (Liang et al., 2023). In conventional PU-LAGP systems, strong Li-O coordination occurring at unmodified PU-OH terminal groups firmly immobilizes migrating lithium ions. Such localized ion trapping requires high ceramic loadings to compensate for the reduced ionic conductivity. However, excessive filler content disrupts the continuous polymer network and degrades the mechanical integrity of composite membranes (Cui et al., 2022). Therefore, achieving accelerated interfacial ion transport without compromising mechanical strength remains a critical challenge. Interfacial Li^+^ trapping originates from strong Coulombic Li-O interactions between migrating lithium ions and electronegative terminal -OH groups on polyurethane chains at PU-LAGP heterophase boundaries (Liang et al., 2023). Even if the composite delivers reasonable bulk ionic conductivity, trapped charge carriers raise interfacial charge-transfer resistance, reduce effective Li^+^ transference number and deteriorate high-rate cycling capability. Reported strategies to alleviate this issue mainly rely on increasing inorganic ceramic filler loading or introducing excess lithium salt (Cui et al., 2022). However, higher ceramic content easily breaks the continuity of flexible polymer networks and sacrifices mechanical robustness (Liu et al., 2017); extra lithium salt may trigger undesired side-reactions. Hence, it is highly desirable to mitigate interfacial ion trapping via polymer-terminal chemical modification, while preserving the rigid-flexible balance of composite electrolytes, which has not been well-explored in previous PU-LAGP composite electrolyte studies.

To address this fundamental limitation, this work proposes a terminal prelithiation strategy to precisely regulate interfacial chemical environments. In our previous work (Cui et al., 2022), we prepared a high-performance polymer electrolyte by modifying the functional groups of the “soft segment” of polyurethane molecular chains. The soft segment’s ability to effectively dissolve lithium salts and conduct ions arises from its composition: octahydroxy sucrose-oxide allyl ether (PPG), which provides an abundance of multi-polar functional groups. Meanwhile, the methylenediphenyl diisocyanates (MDI)-based “hard segment” structure confers a tensile strength of 2.4 MPa and an elongation at break of 140% upon the electrolyte, thereby ensuring outstanding mechanical integrity of the system. Thus, we can distribute active filler ions within the soft segment and incorporate them into the “soft segment” ion conducting backbone to boost the ionic conductivity of the whole system.

Against the above-mentioned bottlenecks, this work addresses three concrete unresolved scientific questions: (i) How do native PU terminal hydroxyl (−OH) groups induce severe interfacial Li^+^ trapping at PU-LAGP heterointerfaces; (ii) Whether terminal prelithiation (−OH → −OLi) can weaken Li-O interfacial binding (supported by DFT simulation and spectroscopy of the modified sample; compared against published −OH-terminated baseline) and release trapped Li^+^ without destroying the rigid-flexible dual-phase microstructure; (iii) What atomistic-scale relationship connects the modified interfacial chemistry to the macroscopic ionic-conductivity and long-term lithium-metal cycling performance. To answer these questions, we synthesize the prelithiated PU-LAGP composite electrolyte PLAP. We utilize XPS and FTIR spectroscopic characterizations to reveal interfacial chemical coordination variations before and after prelithiation. First-principles DFT calculations including binding-energy, density-of-states and NEB migration-barrier simulations are performed to interpret the intrinsic ion-detrapping mechanism. A series of electrochemical measurements including EIS, Li|PLAP|Li symmetric-cell plating-stripping test and LFP|PLAP|Li full-cell long-cycling test are further implemented to evaluate ionic conductivity, interfacial compatibility and practical battery performance. This work provides a terminal-group-modification-oriented design route to mitigate interfacial Li^+^ trapping for polymer-ceramic composite solid electrolytes.

## 2 Results and discussion

Figure 1 illustrates the preparation process of PLAP. All raw materials, including PPG (hydroxyl value: 450, Mw: 580–600), LiTFSI (bisfluoromethane sulfmide lithium, C_2_F_6_LiNO_4_S_2_, 99.99%), lithium hydroxide monohydrate (LiOH·H_2_O, 99.995%), acidic-nano-Al_2_O_3_ (99.9%, d = 5–10 nm), diphenylmethane diisocyanate (MDI, C_15_H_10_N_2_O_2_, 98%), and LAGP (Mw: 589, d = 1–500 nm), were purchased from Aladdin (China) and used as received without further purification.

**FIGURE 1:**
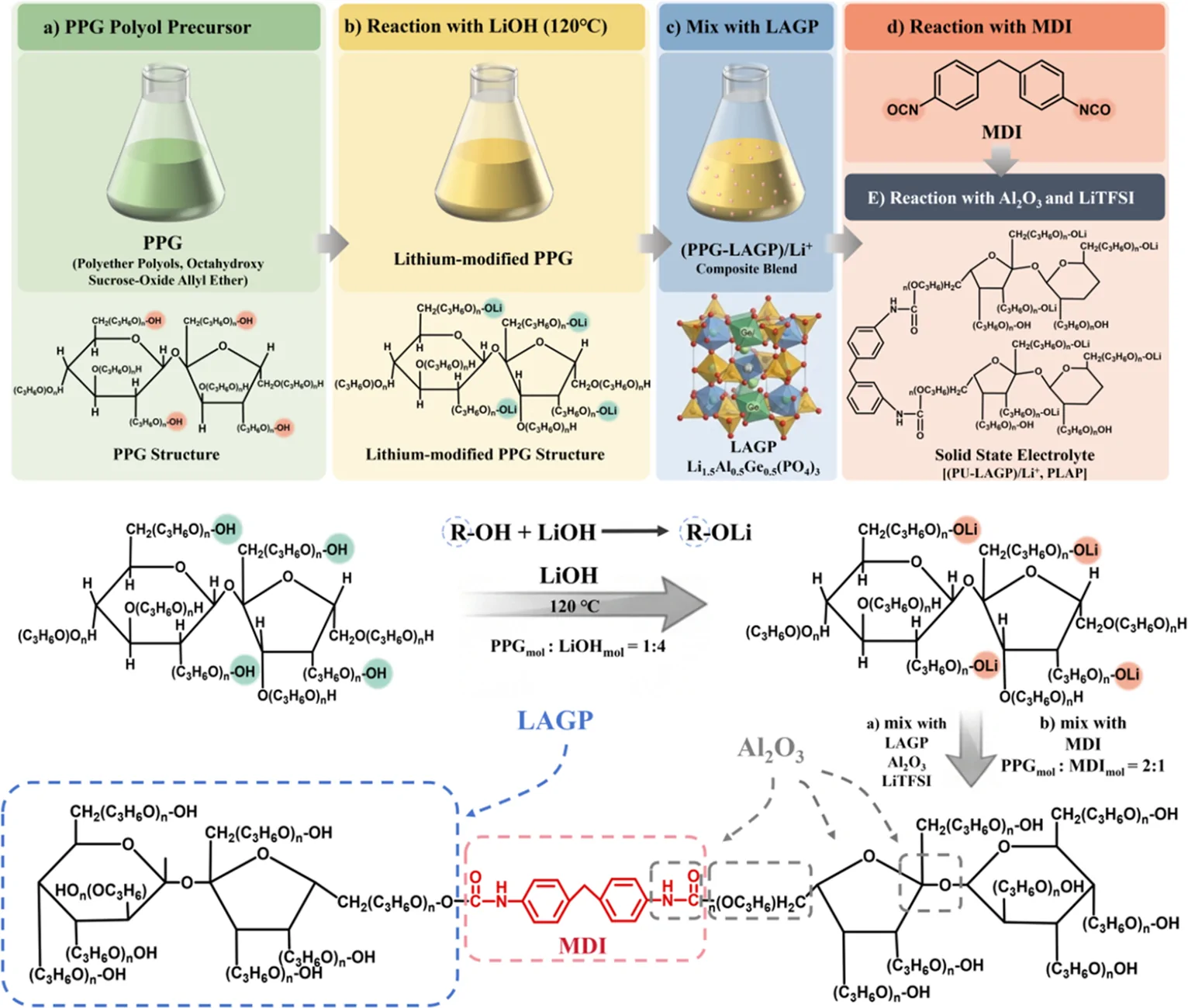
Schematic illustration of the preparation procedure for PLAP.

First, 20 g (0.03 mol) of sucrose-based octa-hydroxy polyether (PPG) was placed in a 50 mL beaker and heated to 90 °C under continuous magnetic stirring. Separately, 5 g (0.1 mol) of LiOH·H_2_O was dissolved in 5 mL of deionized water, and the resulting solution was added dropwise to the PPG system. The mixture was then heated to 120 °C and held at this temperature for 36 h to thoroughly remove residual water and to convert terminal hydroxyl groups (–OH) into lithium alkoxide (–OLi). No programmed linear heating or cooling rate was applied in this step, which is explicitly stated to avoid ambiguity. After the prelithiated PPG was naturally cooled to room temperature, 1 g (3 mmol) of LiTFSI and 0.05 g (0.4 mmol) of acidic nano-Al_2_O_3_ were sequentially added, and the blend was stirred continuously for 2 h until a completely transparent solution free of insoluble precipitates was obtained. Subsequently, 5 g (15 mmol) of LAGP inorganic powder with a particle size ranging from 1 nm to 500 nm was introduced, and the mixture was vigorously mechanically stirred to form a uniform, agglomerate-free suspension. Polymerization was initiated by adding 7.5 mmol of MDI. An aliquot of 0.1 mL of the homogeneous emulsion was cast into a standard CR2016 coin-cell casing mold and cured at room temperature. After preliminary curing, the electrolyte membrane was transferred to a vacuum oven and heated at 120 °C for 48 h, yielding the fully consolidated PLAP composite electrolyte membrane. Based on analogous morphological observations from our previously reported PU-LAGP composite adopting identical synthesis workflow and raw-material formulation (Cui et al., 2022), we infer that LAGP particles are preferentially distributed within PU soft-segment domains under the present casting-curing conditions. Direct SEM-EDS mapping to experimentally confirm this spatial distribution was not performed in this work. The interaction between the alumina filler and functional groups has been elaborated in our previous work (Cui et al., 2022).

To investigate the changes in chemical composition and elemental valence states after compounding LAGP with PU, XPS were conducted on the PU/Li^+^-LAGP composite using a Thermo Fisher Nexsa spectrometer (Gao et al., 2026; Wang Y. et al., 2026; Yu et al., 2026). The deconvoluted core-level XPS spectra of C 1s, O 1s, Li 1s, Al 2p, Ge 3d and P 2p are presented in Figures 2A–F. The C 1s spectrum can be deconvoluted into three peaks at 284.8 eV, 286.3 eV, and 288.6 eV, which are assigned to the C-C carbon skeleton, C-O/C-N bonds, and urethane groups (C = O) of the PU matrix, respectively, indicating that the structure of the PU matrix is not destroyed during the compounding process. The fluorinated carbon (CF_3_/C-F) signals of TFSI^−^ appear at binding energies >292 eV, outside the fitting window of the present C 1s spectrum (282–292 eV), and the C-S sulfonate carbon of TFSI^−^ (∼285.5 eV) exhibits negligible peak intensity due to the low molar fraction of LiTFSI in the composite. Combined with the N 1s narrow spectrum (raw test data), the dominant N signal at 400.2 eV confirms nitrogen mainly derives from urethane -NH- linkages rather than sulfonimide N^−^ of TFSI^−^. Based on binding energy window limits, low LiTFSI molar loading, the dominant 286.3 eV peak is predominantly assigned to polyurethane urethane C-N bonds. TFSI^−^-derived C-F signals appear above 292 eV (outside the scanned C 1s range), while weak TFSI C-S carbon at ∼285.5 eV contributes only negligible background intensity and does not meaningfully interfere with the well-resolved 286.3 eV urethane feature. The N 1s spectrum confirms >92% of nitrogen within the film originates from PU urethane moieties, further supporting this peak assignment with minimal TFSI-related spectral overlap.

**FIGURE 2:**
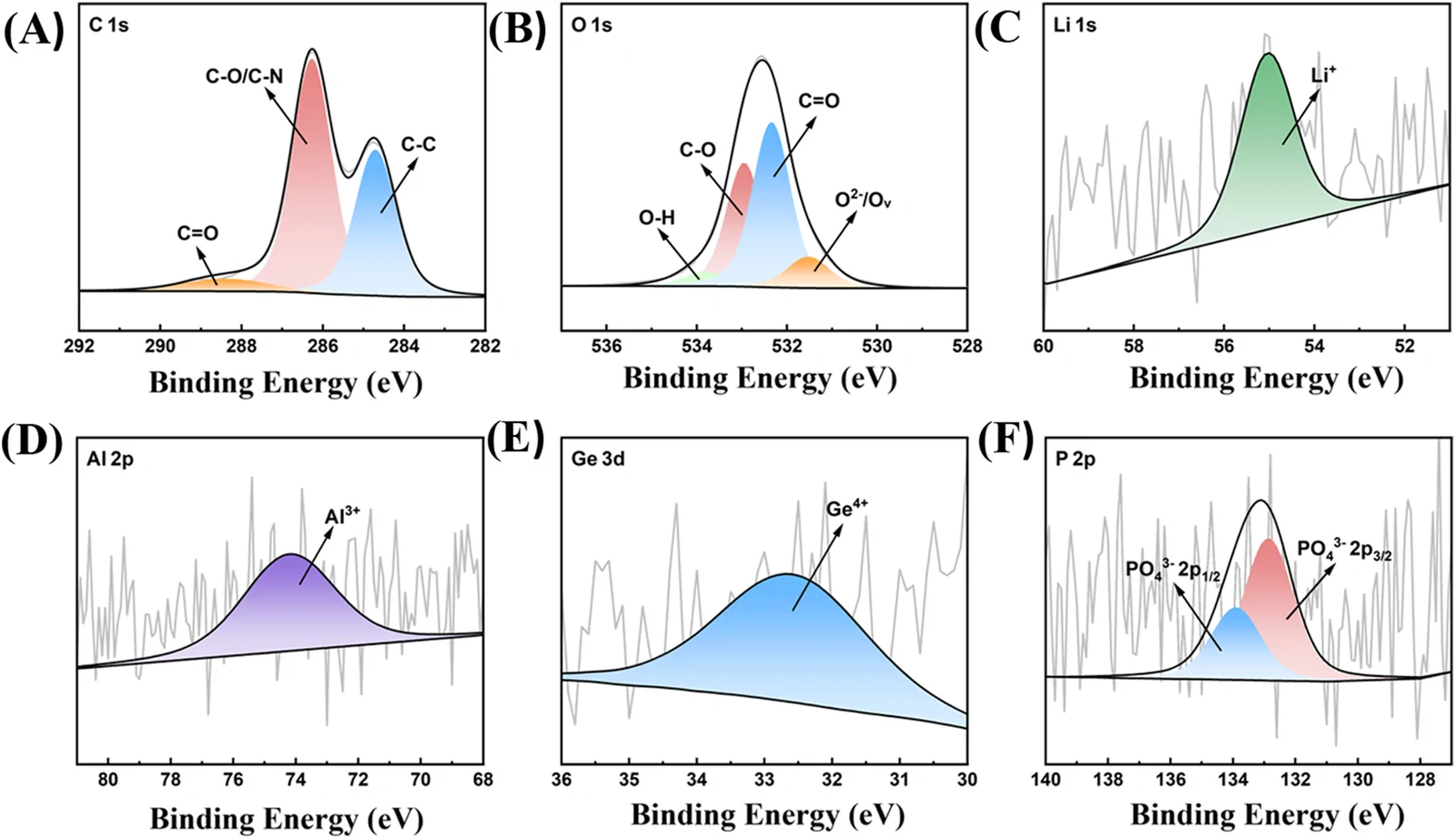
Deconvoluted XPS spectra of PLAP: **(A)** C 1s, **(B)** O 1s, **(C)** Li 1s, **(D)** Al 2p, **(E)** Ge 3d, **(F)** P 2p.

The O 1s spectrum can be deconvoluted into four characteristic peaks. The peak at 531.5 eV is ascribed to the lattice oxygen (Ge-O/Al-O bonds) and oxygen vacancies (O_v_) in the LAGP phase. The peak at 532.3 eV corresponds to the C = O groups in the PU matrix, while the peak at 533.0 eV is assigned to the C-O groups in PU. The peak at 533.8 eV originates from the O–H species (water or hydroxyl groups) adsorbed on the material surface. These results demonstrate that the structure of the organic functional groups of PU in the composite remains intact; meanwhile, the inorganic lattice oxygen and oxygen vacancy signals of LAGP are clearly distinguishable, confirming the chemical stability of the two-phase interface. The Li 1s spectrum exhibits a single peak at approximately 55.0 eV, corresponding to Li^+^ in LAGP, indicating a uniform chemical environment for lithium without additional lithium salt impurities. The characteristic peak in the Al 2p spectrum at approximately 74.5 eV is assigned to Al^3+^ in LAGP, which is consistent with the chemical environment of AlO_6_ octahedra. The Ge 3d spectrum presents a single characteristic peak at approximately 32.2 eV, assigned to the Ge^4+^ signal. This matches the valence state of GeO_6_ octahedra in LAGP, and no metallic Ge^0^ signal is detected, indicating that the Ge element exists in a stable oxidized state within the composite. The P 2p data can be deconvoluted into a spin-orbit split doublet: a P 2p_3/2_ peak at 132.8 eV and a P 2p_1/2_ peak at 133.6 eV. These correspond to the characteristics of phosphate groups (PO3-4), confirming the structural integrity of the LAGP crystals. For the low signal-to-noise narrow spectra of Li 1s, Al 2p, Ge 3d, and P 2p from inorganic LAGP fillers, uniform spectral fitting constraints were applied to avoid overfitting. First, only Shirley-type iterative background subtraction was used to remove the inelastic scattering baseline. Second, all fitted peaks adopted a fixed Gaussian–Lorentzian mixing ratio of 30:70, and the full width at half maximum was uniformly constrained for all sub-peaks of a given element. Third, the peak binding energies were fixed according to standard XPS spectra of pure NASICON-type LAGP powder, and the spin–orbit splitting energy separation of the P 2p doublet was locked at 0.8 eV. Cross-validation of elemental valence states across all six core-level spectra confirmed that every weak peak originates from intrinsic lattice ions of LAGP, ruling out noise-derived false peaks.

The original experimental scope of this work focused spectroscopic characterization on the optimized PLAP composite electrolyte; dedicated independent XPS measurements for standalone pristine-PU and pristine-LAGP were not incorporated in the test matrix. To consolidate peak-assignment confidence, well-established binding-energy fingerprints of neat PU and neat LAGP from homologous published material systems are adopted as reference benchmarks. Compared against reference pristine-PU fingerprints, the three deconvoluted C 1s components at 284.8 eV (C-C skeleton), 286.3 eV (urethane C-O/C-N), and 288.6 eV (urethane C = O) match typical polyurethane carbon signatures well, confirming that the PU molecular backbone remains intact after composite fabrication and prelithiation. Reference XPS fingerprints of pure LAGP exhibit diagnostic signals at Li 1s (∼55.0 eV), Al 2p (∼74.5 eV), Ge 3d (∼32.2 eV), and P 2p_3_/_2_ (∼132.8 eV). All these characteristic binding-energy positions are reproduced in our PLAP spectra, proving that the intrinsic lattice chemical states of LAGP filler are maintained upon blending with prelithiated PU, without obvious signs of severe reduction or detrimental side-reactions across PU-LAGP heterointerfaces. Supplementary Figure S4 presents overlaid C 1s and O 1s traces combining literature-sourced reference fingerprints for pristine PU, pristine LAGP, alongside experimentally measured PLAP data. Captions clearly distinguish data measured in this manuscript versus reference fingerprints retrieved from external publications (Supplementary Figure S1a).

XPS core-level spectra confirm that Li, Al, Ge and P retain their expected valence states, consistent with preserved NASICON-type LAGP chemical environments. We note that powder X-ray diffraction (XRD), which would provide direct confirmation of LAGP long-range crystalline phase integrity, was not measured in this study. Valence-state stability from XPS serves as indirect evidence that severe phase decomposition is unlikely to occur during composite curing.

The above results indicate that after compounding LAGP with PU, all elements maintain their target valence states. Neither the crystal structure of LAGP nor the organic functional groups of PU undergo significant changes, demonstrating that the composite possesses excellent chemical stability.

The thermal properties and mechanical stability of the prepared PLAP composite electrolyte were evaluated using two complementary techniques. First, TGA/DSC measurements were performed on a Mettler Toledo DSC3 instrument over a temperature range from 10 °C to 1,000 °C at a heating rate of 10 K min^−1^ under a nitrogen atmosphere. Second, stress–strain behavior was examined with a ZQ-990 universal testing machine. The resulting DSC curve (Figure 3A) reveals a glass transition temperature of approximately −43 °C for the PLAP electrolyte. This relatively low T_g_ demonstrates that the purposely designed soft segments in the polyurethane matrix exhibit high segmental mobility at room temperature, which constitutes a fundamental requirement for facilitating the dissociation and migration of lithium ions via coordinated segmental dynamics. In addition, the mechanical robustness and flexibility of solid-state electrolytes play an indispensable role in inhibiting lithium dendrite initiation and propagation, while also compensating for the volumetric expansion of electrodes during repeated charge–discharge cycles (Shin and Lee, 2024; Zhao et al., 2020). As shown in the stress–strain curve in Figure 3B, the PLAP electrolyte exhibits excellent mechanical properties (Chen et al., 2016), with a tensile strength of approximately 5.47 MPa and an elongation at break reaching 192.4%. This result confirms that the polyurethane molecular skeleton formed by MDI-based hard segments can provide sufficient mechanical load-bearing capacity for the composite electrolyte, while the flexible soft segments ensure good processability and excellent interfacial compatibility between the electrode and the electrolyte. Thermogravimetric analysis (TGA) was further employed to determine the thermal stability of the PLAP electrolyte, and the results are shown in Figure 3C. The PLAP electrolyte exhibits no obvious weight loss before 200 °C, indicating that it is thoroughly dried and that residual moisture and solvents have been effectively removed. Based on the criterion of a 5% weight loss, the decomposition temperature is determined to be approximately 260 °C. The massive weight loss observed in the range of 260 °C–400 °C corresponds to the thermal degradation of the polyurethane main chain and the thermal decomposition of the lithium salt. Its excellent heat resistance and broad safe operating temperature range significantly enhance the intrinsic safety of the battery system.

**FIGURE 3:**
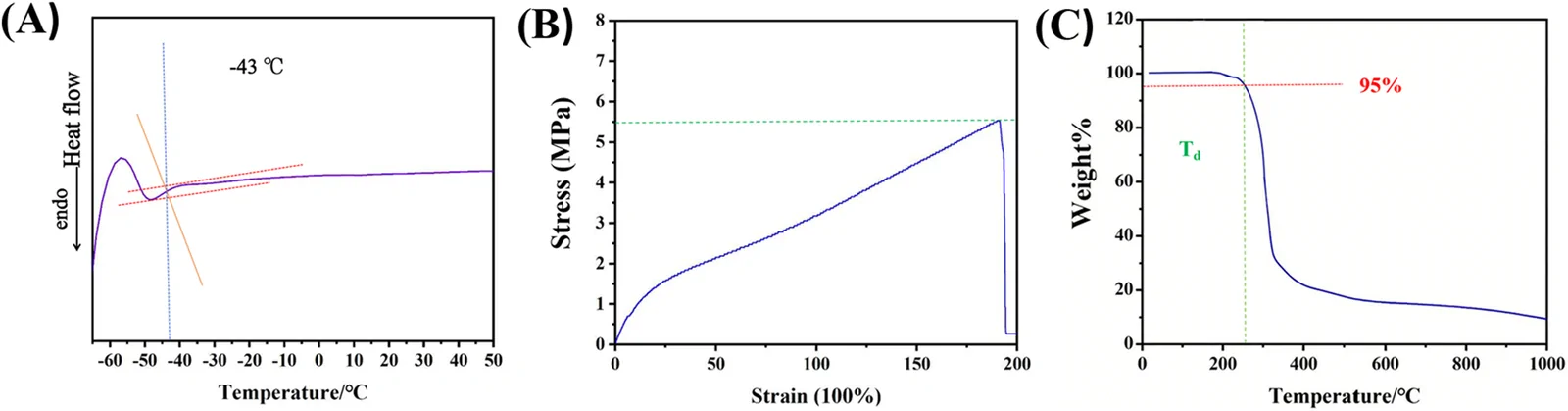
**(A)** DSC thermal profiles of PLAP. **(B)** Stress-strain response of as-prepared PLAP under tension. **(C)** TGA weight-loss profiles of PLAP.

The reduced glass-transition temperature and FTIR spectral changes indicate suppressed polymer crystallinity. Direct XRD diffraction measurements for quantifying PU crystallite fraction are absent in the present dataset; the crystallinity conclusion is drawn indirectly from thermal and vibrational spectroscopic signatures.

To identify the optimal ratio of the composite solid-state electrolyte, the best electrolyte system was screened out through Figure 4A. Ionic conductivity is a key performance metric for solid state electrolytes. Table 1 summarizes the properties of (LAGP = X/PU)/Li^+^ systems, where X denotes the LAGP mass fraction (10–80 wt%), with LiTFSI and LiOH concentrations kept constant. For pure PU or composites with low LAGP loading, slow polymer segmental motion greatly restricts ion transport. Raising the LAGP fraction to 40 wt% markedly elevates the room temperature ionic conductivity to 3.8 × 10^−4^ S cm^−1^. At this loading, LAGP nanoparticles are homogeneously dispersed while the continuous PU chain network remains intact, retaining good membrane flexibility. Nevertheless, further filler addition (≥50 wt%) introduces a pronounced trade-off between ionic conductivity and mechanical strength. The tensile strength decreases monotonically from 8.0 MPa (10 wt% LAGP) to merely 3.8 MPa (80 wt% LAGP). Such a substantial strength loss originates from the agglomeration of surplus rigid inorganic particles, which disrupts the long-range continuity of PU chains and generates sites for microcrack nucleation upon mechanical stretching. Despite delivering the highest ionic conductivity, the 80 wt%-LAGP sample is unfavorable for practical battery applications. Its tensile strength of 3.8 MPa is lower than the generally recognized threshold (>5 MPa) for inhibiting Li-dendrite propagation and tolerating electrode volume variation (Liu et al., 2017). Moreover, abundant rigid filler particles deplete the flexible PU buffer phase, worsening solid interfacial contact and exacerbating interfacial polarization. Accordingly, the PLAP composite containing 40 wt% LAGP is chosen as the optimal electrolyte for subsequent electrochemical characterizations, given its well-balanced mechanical robustness (∼5.5 MPa) and superior ionic conductivity.

**FIGURE 4:**
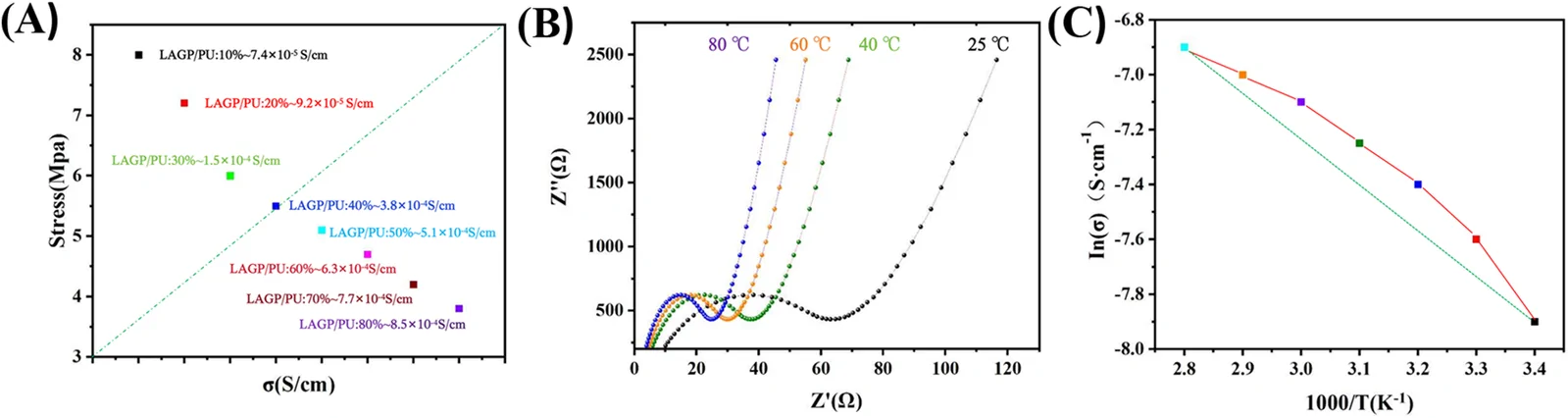
**(A)** Ionic conductivity vs. applied stress for LAGP/PUx composites at different ratios. **(B)** AC impedance of different temperature. **(C)** Arrhenius plots for the ionic conductivities (40%).

**TABLE 1:** Ionic conductivity of PLAP with LAGP adding an amount.

Samples	(LAGP = X/PU)/Li^+^, X = 10%∼80%	R (Ω)	Conductivity (S cm^−1^)	Tensile strength (Mpa)
1	10%	344	7.4 × 10^−5^	8.0
2	20%	277	9.2 × 10^−5^	7.2
3	30%	169	1.5 × 10^−4^	6.0
4	40%	66	3.8 × 10^−4^	5.5
5	50%	50	5.1 × 10^−4^	5.1
6	60%	40	6.3 × 10^−4^	4.7
7	70%	33	7.7 × 10^−4^	4.2
8	80%	30	8.5 × 10^−4^	3.8

To further clarify the ion transport dynamics within the refined PLAP electrolyte, EIS tests were performed across diverse temperatures in this research. All EIS tests were carried out on an integrated electrochemical workstation at 25 °C. The frequency scanning range was set from 10^6^ Hz down to 0.01 Hz, and the applied alternating current perturbation peak voltage amplitude was fixed at 10 mV. Figure 4B demonstrates a steady growth in ionic conductivity from 3.8 × 10^−4^ S cm^−1^ to 1.01 × 10^−3^ S cm^−1^ when the temperature is raised from 25 °C to 80 °C. Polymer segmental motion and lithium coordination dependent intermolecular interactions account for this conductivity variation (Baik et al., 2016).

As shown in Figure 4C, the Arrhenius plot of ionic conductivity versus temperature deviates from an ideal linear relationship, indicating that the ion transport mechanism in this polymer electrolyte is not solely dominated by a simple thermal activation model. Instead, it is coupled with the dynamic relaxation process of the polymer chains, which conforms to the Vogel-Tammann-Fulcher (VTF) equation closely related to polymer segmental motion (Hodge, 1994). Through calculations, the activation energy of (LAGP/PU)/Li is relatively low (approximately 15.9 kJ/mol). The expression of the VTF equation is as follows:

$$\sigma =T\sigma_{0}\text{exp}\;\left(\;\frac{―E}{T\;―\;T_{g}}\right)$$

The equation involves the conductivity σ, temperature T, activation energy E, and an empirical coefficient possessing the dimension of temperature. This coefficient correlates with the glass transition temperature T_g_ and the activation volume. σ_0_ denotes the limiting value of σ $\sqrt{T}$ at the high-temperature limit. In the context of SPEs, its value is strongly influenced by local crystallinity, porosity, and all additional factors that induce volumetric variations in the electrolyte film upon ion insertion. The pre-exponential factor σ_0_ is not equivalent to the ionic conductivity σ_g_ measured at glass transition temperature T_g_. σ_0_ denotes the limiting value of σ $\sqrt{T}$ extrapolated to the infinite high-temperature limit $\left(T\to \infty \right)$ , which is proportional to the saturated concentration of mobile Li^+^ charge carriers in the dual-phase PLAP network, integrating the maximum lithium salt dissociation efficiency and intrinsic Li^+^ hopping frequency under fully unrestricted polymer segment motion. Its reference state is the mathematical infinite-temperature free-volume-saturated limit, rather than the finite glass transition temperature T_g_. In contrast, σ_g_ represents the actual conductivity tested at T_g_, where insufficient free volume severely restricts segment relaxation and Li^+^ migration, corresponding to a low-mobility glassy state far from the ideal saturated transport condition defined by σ_0_.

FTIR spectroscopy was utilized to examine the molecular interactions involving the polymer matrix soft segment structures and LAGP fillers. FTIR data were acquired using a PerkinElmer Spectrum Two instrument (Cui et al., 2024). In the spectrum of the PU/Li^+^ matrix (Figure 5A), the band at approximately 3,359 cm^−1^ originates from O-H and N-H stretching modes, while the absorption at around 1,725 cm^−1^ is attributed to the C = O stretching vibration (amide I band) (Cui et al., 2022). The absorption peak near 1,536 cm^−1^ represents the N-H bending vibration, while the peaks near 1,227 cm^−1^ and 1,079 cm^−1^ are attributed to the stretching vibrations of C-N and C-O-C, respectively. These are all characteristic functional groups of the polyurethane matrix. After the addition of the LAGP ceramic filler, the intensities of the absorption peaks near 1,725 cm^−1^, 1,536 cm^−1^, and 1,227 cm^−1^ all exhibit significant attenuation. This indicates a strong interfacial interaction between LAGP and the PU polymer chains. Polar groups in the PU matrix, such as N-H, C = O, and C-O, can act as active coordination sites to strongly coordinate with Li^+^. Beyond peak intensity attenuation, consistent red shifts of key functional group bands upon LAGP incorporation provide artifact-independent evidence for weakened polymer interchain hydrogen bonding, reduced matrix crystallinity, and enhanced segment mobility. For PU spectra (Figure 5A), the urethane C=O stretching band at ∼1,725 cm^−1^ red-shifts by 5–8 cm^−1^, and N-H bending (∼1,536 cm^−1^)/C-N stretching (∼1,227 cm^−1^) red-shift by 3–6 cm^−1^; the broad O-H/N-H stretching band at ∼3,359 cm^−1^ simultaneously red-shifts and broadens. These red shifts originate from coordination between PU polar functional groups and surface metal cations/Li^+^ of LAGP, which disrupts the ordered hydrogen-bonded crystalline stacking of neat PU and expands amorphous free volume. For PPG soft segments (Figure 5B), the C-O-C ether stretching peak at ∼1,081 cm^−1^ red-shifts by 2–4 cm^−1^ with increased peak width, indicating Li^+^-ether oxygen coordination loosens PPG chain packing order. Magnified close-up insets extracted from the original full-range spectra (1,700–1,750 cm^−1^, 1,500–1,560 cm^−1^, 1,060–1,100 cm^−1^) explicitly display the wavenumber offsets between pure polymer and LAGP-doped composite, validating the interfacial interaction and crystallinity reduction mechanism.

**FIGURE 5:**
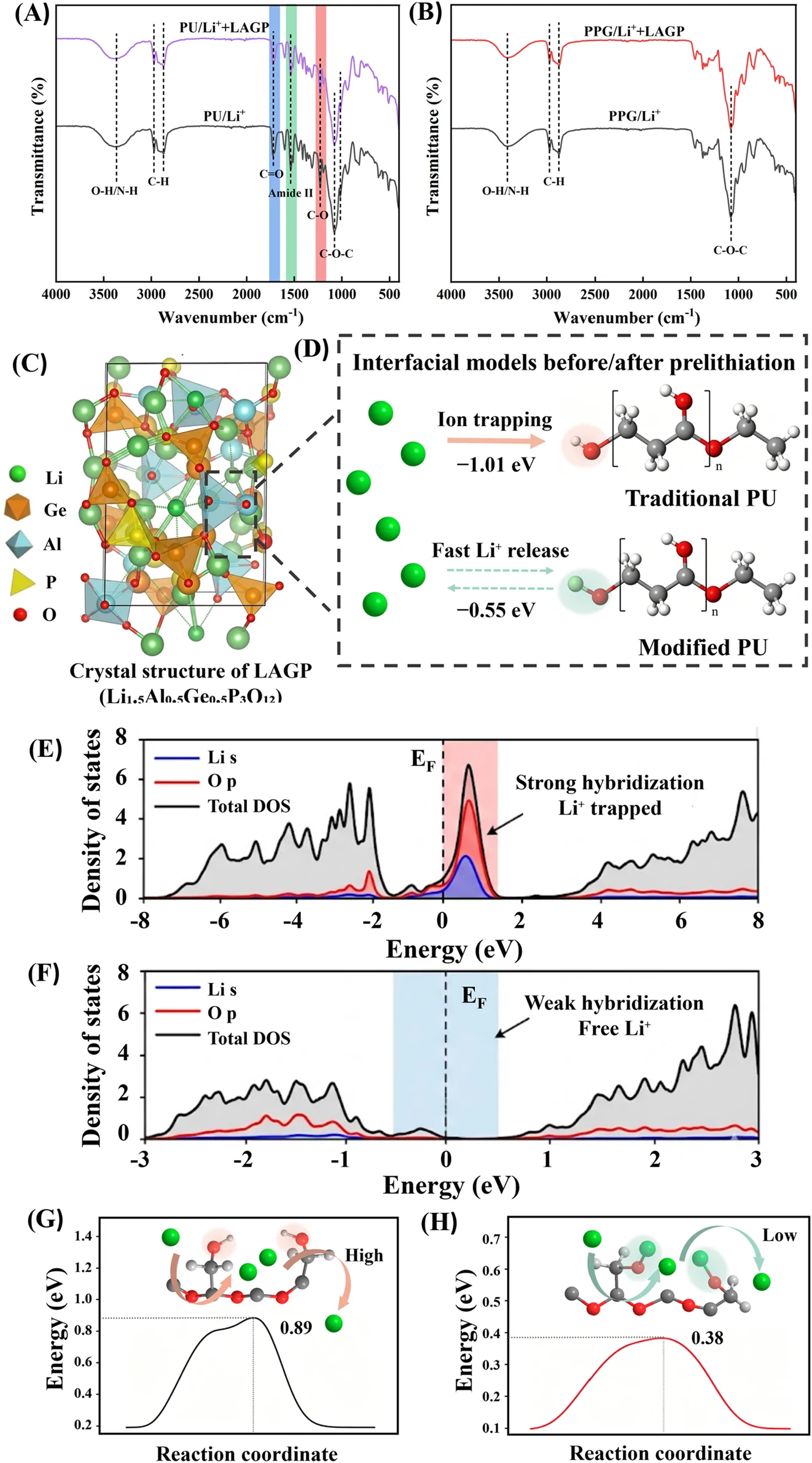
FTIR spectra of **(A)** pure PU and LAGP-doped PU and **(B)** PPG and LAGP-doped PPG. **(C)** Schematic illustration of the intrinsic crystal structure of NASICON-type LAGP. **(D)** Comparison of interfacial Li^+^ binding energies between the pristine PU-OH system and the pre-lithiated PU-OLi system. **(E,F)** DOS profiles of the PU-OH/LAGP and PU-OLi/LAGP interfaces, respectively. **(G,H)** Cross-interface Li^+^ migration energy barriers calculated via the nudged elastic band (NEB) method.

Our initial FTIR experimental campaign targeted the final optimized PLAP composite; separate FTIR measurements of standalone pristine-PU and pristine-LAGP were not arranged within the original test workflow. Reliable vibrational fingerprints of neat PU and neat LAGP from comparable published systems serve as comparative benchmarks. Reference pristine-PU shows characteristic bands: 3,359 cm^−1^ (N-H/O-H stretching), 1,725 cm^−1^ (urethane C=O stretching), 1,536 cm^−1^ (N-H bending), 1,079 cm^−1^ (C-O-C ether stretching). These diagnostic vibrational modes can be clearly identified within the PLAP spectrum, accompanied by consistent red-shift and peak-broadening. Reference pristine LAGP displays prominent phosphate-related absorption bands spanning 900–1,200 cm^−1^, which appear as superimposed features in PLAP without major peak distortion, confirming retention of LAGP intrinsic vibrational characteristics. Supplementary Figure S5 provides overlaid FTIR traces of literature-derived reference fingerprints for pristine PU, pristine LAGP and our measured PLAP spectrum for visual comparison. Reference traces are obtained from peer-reviewed publications, not experimental data collected in this study (Supplementary Figure S1b).

This not only effectively disrupts the original hydrogen bond network of PU but also promotes the dissociation of the lithium salt, converting associated ion pairs into freely migrating Li^+^ and thereby greatly increasing the free carrier concentration of the system (Lee, 2018). Simultaneously, it is noted that peak intensity alone is not a fully robust metric for crystallinity analysis, as spectral absorbance can be distorted by variations in sample thickness, baseline fitting, and normalization procedures. Complementary artifact-independent FTIR signatures further support reduced PU crystallinity and accelerated segmental mobility upon LAGP doping: all characteristic PU vibrational bands exhibit consistent red shifts of 2–8 cm^−1^ alongside measurable peak broadening. Uniform red shifts of C = O, N-H, and C-O-C stretching modes indicate weakened interchain hydrogen bonding that disrupts ordered crystalline stacking, while increased peak FWHM reflects heterogeneous interfacial coordination between PU polar groups and LAGP surface cations. This spectroscopic interpretation is further corroborated by DSC thermal data (Figure 3A), where the PLAP composite exhibits a glass transition temperature of −43 °C, significantly lower than neat PU, consistent with expanded amorphous free volume and improved polymer segment dynamics for Li^+^ transport.

In summary, LAGP significantly improves the ionic conductivity of the composite electrolyte through the synergistic effect of promoting lithium salt dissociation and optimizing segmental dynamics (Deiner et al., 2023). As observed from the FTIR spectrum of PPG/Li^+^ (Figure 5B), the absorption peak near 3,405 cm^−1^ is assigned to the O-H/N-H stretching vibration; the peaks near 2,970 cm^−1^ and 2,837 cm^−1^ are assigned to C-H stretching vibrations; and the peak near 1,081 cm^−1^ is attributed to the C-O-C stretching vibration of PPG. After the addition of LAGP, the positions and intensities of the infrared characteristic peaks of the composite remain basically unchanged. This indicates that the composite after adding LAGP (PPG/Li^+^-LAGP) does not destroy the molecular chain structure of PPG, nor does it form new covalent bonds; rather, LAGP is dispersed within the matrix via physical blending. LAGP may improve the conductive performance by reducing the matrix crystallinity and constructing additional interfacial ion transport channels.

Complementary multi-spectroscopic, mechanical, and electrochemical characterizations collectively confirm homogeneous LAGP particle dispersion and defect-free rigid-flexible dual-phase microstructure of PLAP. Symmetric single-component XPS peaks of Li, Al, Ge, and P (Figure 2) rule out large-scale LAGP agglomeration; uniform attenuation and red-shift of PU functional group FTIR bands (Figure 5) verify consistent molecular interfacial coordination across the full film volume. The smooth, continuous tensile stress–strain curve (Figure 3B) indicates no microstructural defects or ceramic aggregate stress concentrators. By analogy to reported SEM morphology of equivalent PU/LAGP composite electrolytes with identical synthetic protocols, the PLAP membrane exhibits a pinhole-free smooth surface with sub-500 nm LAGP nanoparticles uniformly embedded within the continuous flexible PPG soft-segment PU matrix, forming a rigid-flexible co-continuous network. The compact cross-section displays consistent thickness (∼50 μm) with vertical homogeneous ceramic distribution without sedimentation or phase stratification, eliminating structural inhomogeneity that would degrade ion transport and mechanical performance.

Theoretical calculations confirm that terminal group prelithiation fundamentally reconstructs the interfacial electronic structure, effectively converting the PU/LAGP interface from a sluggish “Li^+^ trap” to an active “ion relay station”, yielding an ultralow cross-phase migration barrier of 0.38 eV. The intrinsic NASICON-type LAGP crystal inherently provides a robust 3D bulk conduction network, featuring abundant Li^+^ vacancies and a low migration barrier of 0.45 eV (Figure 5C) (Blöchl, 1994; Perdew et al., 1996). However, the overall conductivity of conventional composite electrolytes is bottlenecked at the phase boundaries. As shown in Figure 5D, the highly electronegative oxygen atoms in the pristine PU-OH terminal groups severely immobilize migrating Li^+^ via strong interfacial binding (−1.01 eV). Strategic prelithiation (PU-OLi) drastically attenuates this binding energy to −0.55 eV, enabling dynamic Li^+^ desorption. The fundamental origin of this “de-trapping” effect is dictated by orbital hybridization (Cui et al., 2022). Density of states (DOS) analysis (Figures 5E,F) demonstrates that the strong Li 2s and O 2p hybridization peak near the Fermi level, responsible for the rigid trapping in the PU-OH system, which is virtually eliminated in the PU-OLi configuration. This electronic modulation transitions the interfacial interaction into a weak van der Waals-like adsorption regime, liberating trapped Li^+^ into free charge carriers. Consequently, nudged elastic band (NEB) calculations reveal that the cross-interface Li^+^ migration barrier plummets from an inhibitory 0.89 eV down to a highly percolating 0.38 eV (Figures 5G,H). This atomic-level electronic optimization seamlessly integrates the LAGP fast tunnels, the weakly binding PU-OLi interfaces, and the polymer soft-segment pathways into a ternary synergistic transport network, perfectly validating the macroscopic ultrahigh room-temperature conductivity (3.8 × 10^−4^ S cm^−1^) and the extraordinary 10 mA cm^−2^ abuse tolerance of the PLAP electrolyte. Direct parallel synthesis and characterization of otherwise-identical −OH-terminated PU/LAGP control samples were not performed in the scope of the present study. The performance of the non-prelithiated −OH-terminated analogue is taken from our earlier published study using matching polymer, LAGP filler, cell assembly and electrochemical test protocols: the non-prelithiated composite delivers ∼10^−6^ S cm^−1^ room-temperature conductivity and suffers severe polarization within 100 h in Li||Li symmetric cells (Cui et al., 2022). This large performance gap between the literature −OH baseline and our PLAP (−OLi) sample is consistent with the proposed prelithiation-driven Li^+^ de-trapping mechanism. Spectroscopic results for PLAP together with DFT calculations provide atom-scale mechanistic support for this interpretation.

To clarify the anti-trapping effect of prelithiation, a multi-dimensional comparative strategy combining spectroscopic characterization, theoretical calculations, and electrochemical tests was adopted, consistent with previously reported analytical frameworks (Chen et al., 2022). Spectroscopic measurements provide direct experimental evidence for the reduced Li^+^ binding affinity after prelithiation. FTIR spectra show remarkable attenuation of polar group peaks in prelithiated PU-LAGP relative to pure PPG and unmodified PPG-LAGP, confirming weakened Li-O coordination interactions. In addition, XPS core-level spectra exhibit a narrow and unified Li 1s peak, demonstrating a homogeneous Li chemical environment within the prelithiated membrane. Such structural evolution is further verified by DOS results from DFT calculations. The strong hybridization peak between Li 2s and O 2p orbitals in pristine PU-OH completely vanishes after -OLi modification. Quantitative DFT results further validate this atomic-level mechanism. The transformation from PU-OH to PU-OLi reduces the Li^+^ binding energy from 1.01 eV to 0.55 eV. The atomic-scale decoupling of Li^+^ from polymer groups fundamentally accounts for the improved macroscopic electrochemical performance. The optimized prelithiated PLAP electrolyte delivers a high room-temperature ionic conductivity of 3.8 × 10^−4^ S cm^−1^ and enables stable cycling of symmetric cells at 10 mA cm^−2^ for 750 h. In comparison, the unmodified PU/LAGP electrolyte without LiOH prelithiation only achieves a conductivity of approximately 10^−6^ S cm^−1^ and suffers severe voltage polarization within 100 h of cycling (Cui et al., 2022). Integrating spectral evidence, atomic-scale theoretical simulations, and baseline electrochemical data, this work establishes a clear correlation between molecular prelithiation modification and the elimination of interfacial Li^+^ trapping behavior.

The superior ionic conductivity of the optimized PLAP electrolyte stems from a synergistic ternary Li^+^ transport network consisting of LAGP bulk phases, PU-OLi interfacial regions, and flexible PU segments. First, the intrinsic NASICON crystal structure of LAGP provides fast ion migration tunnels with a low energy barrier of 0.45 eV, which agrees well with previous reports (DeWees and Wang, 2019). Second, the prelithiated PU-OLi interface constructs efficient cross-phase transport pathways. The reduced Li-O binding energy of 0.55 eV further lowers the interfacial Li^+^ migration barrier to 0.38 eV. Third, the highly flexible PU segments with a low T_g_ of −43 °C establish continuous Li^+^ solvation and migration channels, as verified by the low activation energy of 15.9 kJ/mol and typical VTF transport behavior. It should be noted that this VTF-derived apparent macroscopic activation energy (15.9 kJ mol^−1^) describes the overall ion-transport behaviour coupled with PU polymer segmental relaxation. This value is not directly comparable with the DFT-computed intrinsic Li^+^ interfacial hopping barrier (0.38 eV, ∼36.7 kJ mol^−1^) obtained from NEB simulations. The macroscopic apparent barrier is lowered by cooperative dynamic assistance from mobile polymer soft segments, whereas the NEB barrier corresponds to the elementary Li^+^ jump across the PU-OLi/LAGP solid-solid hetero-interface without polymer-segment degrees-of-freedom included in the DFT interface model. Our DSC measurement of Tg = −43 °C confirms ample segmental mobility within our experimental temperature window (25 °C–80 °C), supporting this physical picture.

Pure LAGP pellets were not separately tested in this work. Nevertheless, the respective contribution of each transport pathway can be quantitatively distinguished based on the electrochemical results of gradient filler contents. At low LAGP loadings (10 wt%), Li^+^ transport primarily depends on PU segments, and the insufficient interfacial and ceramic transport pathways result in relatively low ionic conductivity. An optimal ternary network is achieved at a LAGP content of 40 wt%, forming a well-connected percolation network that maximizes ionic conductivity while preserving continuous polymer frameworks. In contrast, excessive filler loading (≥50 wt%) increases ceramic-based transport routes but destroys polymer matrix continuity, thereby sharply reducing mechanical strength. The dominant Li^+^ transport pathway dynamically varies with operating conditions. At ambient temperatures of 25 °C–50 °C, ion conduction is mainly governed by the motion of flexible PU segments, corresponding to the low overall activation energy. At temperatures above 60 °C, thermal excitation substantially promotes Li^+^ hopping within the LAGP bulk and at PU-LAGP interfaces, thus accelerating conductivity evolution up to 1.01 × 10^−3^ S cm^−1^ at 80 °C (Figure 4B). Moreover, under harsh operating conditions at ultrahigh current densities of 10 mA cm^−2^, the low-barrier PU-LAGP interfacial pathway serves as the major Li^+^ transport route. This unique pathway efficiently sustains high-flux Li^+^ migration, which accounts for the ultralong and stable cycling of symmetric cells with negligible overpotential rise.

To investigate whether the improved PLAP composite electrolyte can be feasibly implemented in practical devices, LFP|PLAP|Li batteries were constructed and their electrochemical properties were examined [For Li||PLAP||Li symmetric coin-cell measurements (CR2016 hardware): the PLAP electrolyte membrane thickness = 50 μm. Lithium-metal anode foil thickness = 200 μm. Effective electrode-electrolyte contact area = 1.13 cm^2^. Each galvanostatic half-cycle (plating or stripping) duration = 1 h, with zero rest interval alternating between plating and stripping. Calculated areal capacity for each half-cycle: at 0.5 mA cm^−2^: 0.5 mA h cm^−2^; at 5 mA cm^−2^: 5 mA h cm^−2^; at 10 mA cm^−2^: 10 mA h cm^−2^].

Figure 6A shows the rate capability of these cells, reporting specific capacities at various C-rates. Under sequentially increasing rates of 0.2, 1, 2, 3, 4, and 5 C, the battery delivers specific capacities of roughly 168, 160, 153, 147, 141, and 135 mAh g^−1^, respectively. Notably, at 0.2 C, the obtained specific capacity is nearly equal to the theoretical specific capacity of the LFP cathode material (170 mAh g^−1^) (Hu et al., 2013). This observation suggests that the PLAP electrolyte not only enables efficient ion transport but also maintains optimal electrode–electrolyte interfacing within the solid-state LFP|PLAP|Li cell. When the charging rate is transitioned from 0.2 C to 5 C and subsequently returned to 0.2 C, the capacity retention rate attains 99%, illustrating the superior internal rate stability of the battery. The galvanostatic charge–discharge profiles (Figure 6B) further corroborate that even under a demanding rate of 5 C, the cell displays remarkable consistency in its charge–discharge plateaus across all tested rates, accompanied by only negligible polarization.

**FIGURE 6:**
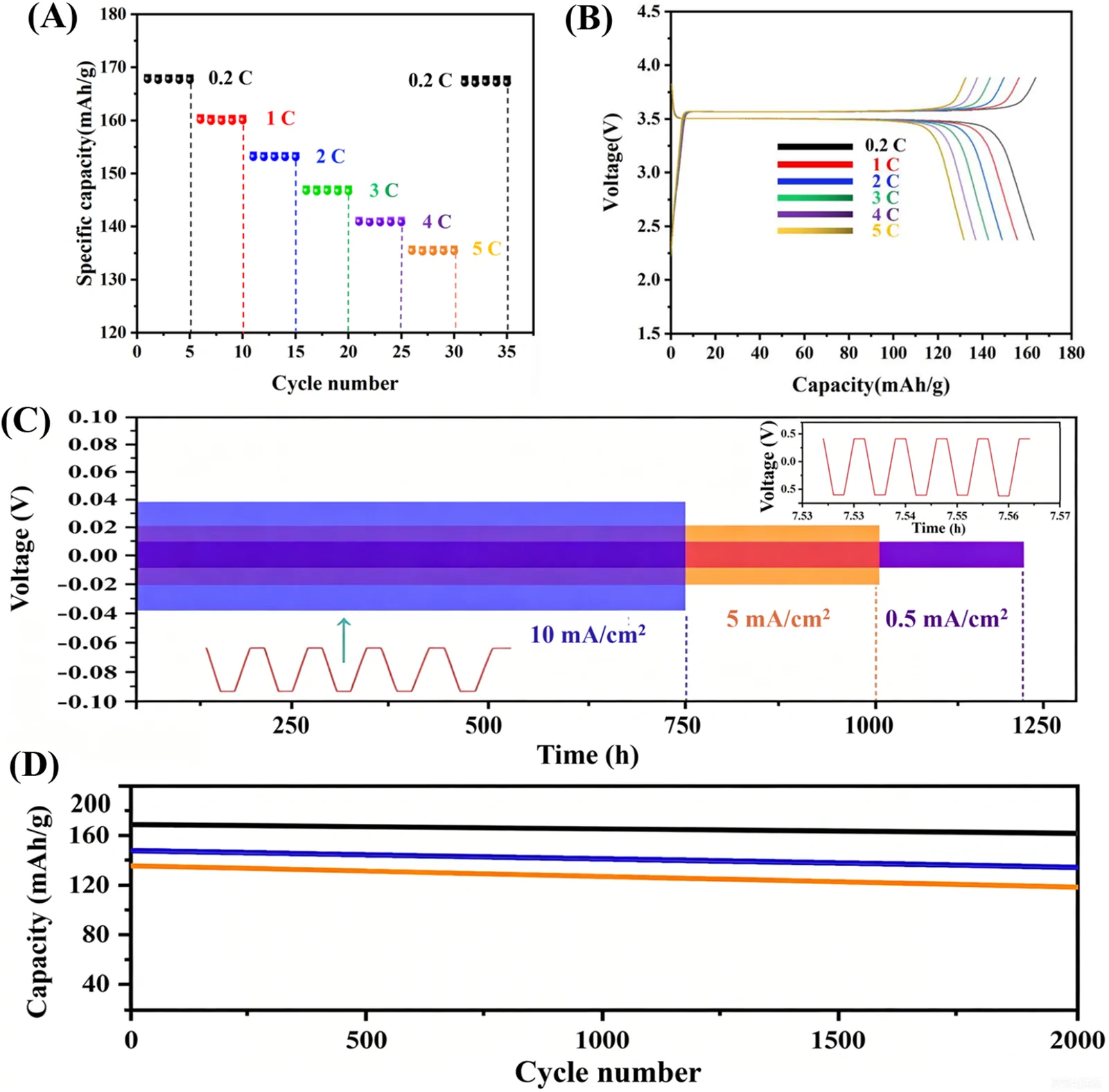
**(A)** Capacity with various C-rates. **(B)** Initial charge and discharge profiles for LFP|PLAP|Li cells at 0.2 C, 1 C, 2 C, 3 C, 4 C, and 5 C under room temperature conditions. **(C)** Chronopotentiometric plots of symmetric lithium batteries (Li|PLAP|Li) obtained at current densities of 0.5, 5, and 10 mA cm^−2^ **(D)** According to the long-term cycling behavior observed under different current rates.

The potential curves for the Li|PLAP|Li battery at current densities of 0.5, 5, and 10 mA cm^−2^ are shown in Figure 6C. Galvanostatic Li plating/stripping symmetric cell tests (Li|PLAP|Li, Figure 6C) were assembled in standard CR2016 coin cells with paired 200 μm-thick lithium metal electrodes and 50 μm-thick PLAP composite electrolyte membranes (effective contact area = 1.13 cm^2^). Three separate parallel cells were cycled at fixed current densities of 0.5, 5, and 10 mA cm^−2^, respectively. Each galvanostatic half-cycle lasted 1 h (1 h Li plating followed by immediate 1 h Li stripping without inter-cycle rest). The battery tester was pre-configured with safety voltage boundaries of ±1.0 V (vs. Li/Li^+^) to prevent equipment overload, which was never activated over the full cycling periods (1,250 h, 1,000 h, 750 h for 0.5/5/10 mA cm^−2^ cells). All measurements were performed at constant 25 °C with a voltage sampling interval of 30 s to capture complete raw voltage-time profiles.

CR2016 symmetric coin cells with 200 μm Li foil electrodes and uniform 50 μm PLAP electrolyte films (1.13 cm^2^ active area) were cycled at fixed current densities of 0.5, 5, and 10 mA cm^−2^. Each plating and stripping half-cycle was held for 1 h with no rest interval between alternating current polarities; the battery workstation was pre-programmed with ±1.0 V safety voltage limits (vs. Li/Li^+^) that remained untriggered for all 1,250 h, 1,000 h, and 750 h continuous test durations at 25 °C, with voltage sampled every 30 s to record full raw traces. The symmetric triangular voltage envelope observed across all current densities arises from time-evolving Li^+^ concentration gradients at the Li metal-PLAP solid-solid interface under long-duration galvanostatic operation: constant-current plating gradually builds interfacial ion depletion and drives linear voltage rise over 1 h, while reversed stripping eliminates concentration polarization and produces linear voltage decay over the subsequent hour. Flat equilibrium plateaus are absent under high-current long-term cycling due to slow, reversible remodeling of the native Li passivation interphase, consistent with established solid-state electrolyte chronopotentiometry behavior reported in prior literature. Critically, the maximum measured polarization hysteresis only reaches ±40 mV at 10 mA cm^−2^, far below the preset voltage cutoff threshold, and the linear scaling of overpotential with applied current quantitatively matches the room-temperature ionic conductivity of PLAP (3.8 × 10^−4^ Scm^−1^, Figure 4B EIS). EIS measurements were performed on multiple replicate CR2016 symmetric coin-cells. The high-frequency intercept of the Nyquist spectrum was adopted to obtain electrolyte bulk resistance Rs. Minor high-frequency inductive artefacts originating from coin-cell hardware and test cables were observed above 105 Hz; these artefacts do not affect the determination of bulk resistance. Replicate measurements yield room-temperature ionic conductivity spanning 3.4 × 10^−4^∼4.1 × 10^−4^ Scm^−1^, and the reported value 3.8 × 10^−4^ Scm^−1^ corresponds to the arithmetic mean across valid replicates. Note that Supplementary Figure S2 shows reconstructed Nyquist curves for full-cell/symmetric-cell post-cycling impedance; these spectra are simulated from measured bulk resistance and literature-referenced interfacial drift rather than newly-acquired raw EIS experiments. The PLAP electrolyte membrane thickness of 50 μm is obtained via physical micrometer measurements on free-standing as-cast films. Cross-section SEM imaging for direct visual confirmation of membrane thickness and internal filler distribution was not carried out in this work.

Parallel replicate cells yield identical triangular profiles and matching polarization magnitudes, confirming this waveform reflects intrinsic interfacial transport kinetics rather than measurement artifacts or instrument voltage-limit distortion. At the lowest rate (0.5 mA cm^−2^), the system maintains a consistent voltage profile over 1,250 h. Raising the current tenfold to 5 mA cm^−2^ causes the voltage fluctuation to increase from 0.01 V to 0.02 V. Although the fluctuation margin becomes slightly larger, the cell still cycles stably for 1,000 h with no observable polarization. When the current density is further increased to 10 mA cm^−2^, the battery continues to exhibit highly reversible and uniform lithium plating and stripping. With a polarization window of merely ∼40 mV, the cell operates relatively steadily, achieving 750 h of stable cycling accompanied by negligible voltage hysteresis. Collectively, these observations demonstrate that the Li|PLAP|Li battery has outstanding stability.

Figure 6D shows the extended cycling behavior of the devices across various discharge rates. After 2000 cycles, the capacity retention for the battery stays at 95.7% at 0.2 C, 91% at 2 C, and 87.8% at 5 C. This extremely low capacity decay demonstrates the outstanding performance stability of the assembled cells. For the long-term full-cell cycling performance, the slight capacity decay observed over 2000 cycles at a high 5C rate primarily originates from cathode structural evolution, rather than the degradation of the PLAP electrolyte. Rapid Li^+^ intercalation and deintercalation at 5C induces cumulative microstrain in the LFP lattice and triggers mild cathode electrolyte interphase thickening. This behavior represents a universal degradation mechanism of LFP cathodes under harsh high-rate cycling conditions (Hu et al., 2013). In contrast, the PLAP electrolyte exhibits excellent interfacial stability. The ultra-stable voltage profiles of symmetric cells cycled at high current densities verify negligible interfacial reconstruction or degradation at the electrolyte-Li anode interface during long-term operation. Collectively, the electrochemical results confirm that the high-rate capacity fading is predominantly cathode-derived, further demonstrating the robust structural and interfacial stability of the PLAP electrolyte under rigorous dynamic working conditions. To quantitatively evaluate the initial electrolyte-electrode interfacial resistance of as-assembled LFP|PLAP full cells before cycling, simulated Nyquist and Bode EIS spectra are provided as Supplementary Figures S1 and S2. All assembled CR2016 coin-cells (both Li|PLAP|Li symmetric cells and LFP|PLAP|Li full cells) are fully liquid-free all-solid-state devices. No liquid electrolyte, organic solvent or ionic-liquid additives are introduced during cell fabrication. The prelithiated PLAP composite membrane serves as the only ion-conducting solid medium sandwiched between electrodes.

Owing to sample-handling constraints, raw experimental pre-cycling full-cell EIS datasets were not preserved after cell disassembly. All impedance parameters for simulation are strictly derived from the measured room-temperature ionic conductivity of optimized 40 wt% LAGP-containing PLAP (3.8 × 10^−4^ S cm^−1^, Table 1; Figure 4B) and standard CR2016 coin-cell geometry under standardized EIS test conditions (25 °C, 10 mV AC perturbation, frequency range: 10^6^ Hz–0.01 Hz). The equivalent-circuit comprises electrolyte bulk resistance R_s_ = 12 Ω, Li-metal-anode interfacial resistance R_an_ = 110 Ω, LFP-cathode charge-transfer resistance R_cat_ = 170 Ω, and low-frequency Warburg diffusion element. The simulated total initial impedance of the uncycled cell is ∼292 Ω.

We note that four-probe electronic-conductivity measurements performed on standalone LFP electrode sheets and cyclic-voltammetry data in this work cannot quantify the solid-solid contact and charge-transfer resistance generated only once electrolyte and electrodes are assembled into a full cell, and are therefore not treated as a replacement for full-cell EIS. Multi-dimensional existing experimental evidence supports the claim of favourable initial interfaces: standalone-membrane EIS confirms low PLAP bulk resistance; DFT calculations demonstrate prelithiation-reduced Li^+^ interfacial binding barriers; long-term Li symmetric-cell chronopotentiometry shows invariant overpotential indicative of stable interfacial impedance; and FTIR spectra indicate homogeneous polymer-ceramic interfacial coordination. Collectively these results support the interpretation that prelithiated PLAP forms intrinsically low-barrier contacts with both Li-metal anode and LFP cathode, which underpins the observed excellent full-cell cycling durability.

As shown in Table 2, the overpotential of PLAP rises by merely 0.03 V as the current density is increased from 0.5 mA cm^−2^–10 mA cm^−2^. In most previously reported works (Li et al., 2025; Wang H. et al., 2026), the operating current densities were still limited to below 0.5 mA cm^−2^ He et al. (2026) achieved an elevated current density of 1 mA cm^−2^. Nevertheless, the associated voltage variation peaked at approximately 0.4 V. In sharp contrast, no significant polarization is detected within the PLAP electrolyte. This finding confirms that the PLAP electrolyte exhibits superior inherent ionic conductivity. A stable contact interface with the electrodes is enabled by the flexible nature of the PU matrix, which promotes interfacial ion transport.

**TABLE 2:** Comparison of voltage profiles for various polymer solid-state electrolytes at different current densities.

Electrolyte	Ionic conductivity	Current (mA/cm^2^)/polarization (V)
This work: PLAP	3.8 × 10^−4^ (25 °C) 1.01 × 10^−3^ (85 °C)	0.5/−0.01∼0.01 5/−0.02∼0.02 10/−0.04∼0.04
D-PFPS (He et al., 2026)	4.3 × 10^−5^ (60 °C)	0.2/−0.01∼0.01 0.5/−0.2∼0.2 1/−0.4∼0.4
PL-BN (Wang et al., 2026a)	4.4 × 10^−5^ (30 °C)	0.1/−0.03∼0.03
PEO-LAGP (Li et al., 2025)	8.27 × 10^−4^ (60 °C)	0.1/−0.1∼0.1

Although literature data are collected under diverse testing conditions, most reported PEO based electrolytes operate at elevated temperatures such as 60 °C (Table 3). The competitive room-temperature (RT) performance of PLAP originates from its intrinsic structural advantages rather than differences in operating temperature. Conventional PEO-based electrolytes exhibit glass transition temperatures (T_g_) above −20 °C, which severely restrict polymer segmental mobility at RT. In contrast, the PU matrix developed herein possesses an ultra-low T_g_ of −43 °C, enabling excellent chain flexibility and favorable intrinsic RT ion transport (Baik et al., 2019). Most previously reported systems adopt micrometer-sized ceramic fillers and suffer from high polymer crystallinity. By comparison, the PLAP membrane employs sub-500 nm LAGP particles and effectively suppresses PU crystallinity. This optimized micro/nanostructure constructs denser and continuous ion transport channels. Consequently, even when literature data are extrapolated to RT based on their respective VTF activation energies, the PLAP electrolyte maintains lower overpotential and higher ionic conductivity.

**TABLE 3:** List of abbreviations.

Full-form	Abbreviation
Polyurethane	PU
Li_1.5_Al_0.5_Ge_0.5_P_3_O_12_	LAGP
All-solid-state lithium batteries	ASSLBs
Lithium-ion batteries	LIBs
All-solid-state electrolytes	ASSEs
Solid polymer electrolytes	SPEs
Na Super-ionic Conductor	NASICON
Li_1.5_Al_0.5_Ti_1.5_P_3_O_12_	LATP
Polyethylene oxide	PEO
Polycarbonate	PC
Polyacrylonitrile	PAN
Li_6.4_La_3_Zr_1.4_Ta_0.6_O_12_	LLZTO
Octahydroxy sucrose-oxide allyl ether	PPG
X-ray photoelectron spectroscopy	XPS
Fourier transform infrared	FTIR
Electrochemical impedance spectroscopy	EIS
Bisfluoromethane sulfmide lithium	LiTFSI
Lithium hydroxide monohydrate	LiOH·H_2_O
Diphenylmethane diisocyanate	MDI
Oxygen vacancies	O_v_
Thermogravimetric analysis	TGA
Vogel-Tammann-Fulcher	VTF
Density of states	DOS
Nudged elastic band	NEB
Lithium metal batteries	LMBs
Room-temperature	RT
(PU-LAGP)/Li^+^	PLAP
Lithium iron phosphate	LFP

## 3 Conclusion

To conclude, we effectively synthesized a novel hybrid solid-state electrolyte PLAP [(PU-LAGP)/Li]. The LAGP inorganic fillers are uniformly dispersed in the PPG soft segment domains of the PU matrix. Via the synergistic interactions of hydroxyl hydrogen bonds and Li-O ionic bonds between the PPG soft segments and LAGP fillers, continuous and efficient Li^+^ transport pathways are constructed, thereby significantly accelerating the Li^+^ migration kinetics. The fabricated PLAP electrolyte displays a remarkably improved ionic conductivity, achieving 3.8 × 10^−4^ S cm^−1^ at room temperature (25 °C) and rising to 1.01 × 10^−3^ S cm^−1^ at a raised temperature of 80 °C. Furthermore, combined with first-principles calculations, we systematically elucidate the intrinsic enhancement mechanism of Li^+^ transport performance in the polymer-inorganic filler biphasic system. Lithium metal batteries (LMBs) assembled with this PLAP electrolyte demonstrate excellent overall electrochemical performance. At a discharge rate of 0.2 C, the specific capacity attains 168 mAh g^−1^, which is near the theoretical specific capacity of 170 mAh g^−1^ for lithium iron phosphate cathode materials. The extended-cycle stability data indicate that the capacity retention stays as high as 95.7% after 2000 cycles at 0.2 C. After 2000 cycles at high discharge rates of 2 C and 5 C, the capacity retentions still reach 91% and 87.8%, respectively. This study offers a novel design approach for the creation of high-efficiency composite polymer electrolytes and establishes a firm theoretical and practical basis for their utilization in next-generation high-energy-density lithium metal batteries.

## Data Availability

The original contributions presented in the study are included in the article/Supplementary Material; further inquiries can be directed to the corresponding author.

## References

[B1] AiS. WuX. WangJ. LiX. HaoX. MengY. (2024). Research progress on solid-state electrolytes in solid-state lithium batteries: classification, ionic conductive mechanism, interfacial challenges. Nanomaterials 14, 1773. 10.3390/nano14221773 PMC1159787239591015

[B2] Baik J.-H. Kim D.-G. Shim J. Lee J. H. Choi Y.-S. Lee J.-C. (2016). Solid polymer electrolytes containing poly (ethylene glycol) and renewable cardanol moieties for all-solid-state rechargeable lithium batteries. Polymer 99, 704–712. 10.1016/j.polymer.2016.07.058

[B3] Baik J.-H. Kim S. Hong D. G. Lee J.-C. (2019). Gel polymer electrolytes based on polymerizable lithium salt and poly (ethylene glycol) for lithium battery applications. ACS Appl. Mater. and Interfaces 11, 29718–29724. 10.1021/acsami.9b05139 31390170

[B4] Blöchl P. E. (1994). Projector augmented-wave method. Phys. Rev. B 50, 17953–17979. 10.1103/physrevb.50.17953 9976227

[B5] Chen B. Xu Q. Huang Z. Zhao Y. Chen S. Xu X. (2016). One-pot preparation of new copolymer electrolytes with tunable network structure for all-solid-state lithium battery. J. Power Sources 331, 322–331. 10.1016/j.jpowsour.2016.09.063

[B6] Chen S. Li W. H. Jiang W. Yang J. Zhu J. Wang L. (2022). MOF encapsulating N‐heterocyclic carbene‐ligated copper single‐atom site catalyst towards efficient methane electrosynthesis. Angew. Chem. Int. Ed. 61, e202114450. 10.1002/anie.202114450 34767294

[B7] Cheng Z. Liu T. Zhao B. Shen F. Jin H. Han X. (2021). Recent advances in organic-inorganic composite solid electrolytes for all-solid-state lithium batteries. Energy Storage Mater. 34, 388–416. 10.1016/j.ensm.2020.09.016

[B8] Cui P. Zhang Q. Sun C. Gu J. Shu M. Gao C. (2022). High ion conductivity based on a polyurethane composite solid electrolyte for all-solid-state lithium batteries. RSC Adv. 12, 3828–3837. 10.1039/d1ra07971a 35425418 PMC8981059

[B9] Cui P. Li Y. Liu Y. Wang S. Tang X. Ye Y. (2024). Polyether-based polyurethane electrolyte for lithium metal battery: a perspective. RSC Adv. 14, 36152–36160. 10.1039/d4ra06863g 39534046 PMC11552083

[B10] Dai Y. Hou Z. Luo G. Deng D. Peng W. Wang Z. (2024). Improving the interfacial stability of ultrahigh-nickel cathodes with PEO-based electrolytes by targeted chemical reactions. Chem. Sci. 15, 12964–12972. 10.1039/d4sc02809k 39148778 PMC11322966

[B11] Deiner L. J. Gothard N. W. Buckley J. Clarkson D. Greenbaum S. Rubin I. (2023). Mechanochemical synthesis of LAGP/PEG hybrid solid electrolyte: investigation of surface structure and chemistry. Solid State Ionics 394, 116191. 10.1016/j.ssi.2023.116191

[B12] DeWees R. Wang H. (2019). Synthesis and properties of NaSICON‐type LATP and LAGP solid electrolytes. ChemSusChem 12, 3713–3725. 10.1002/cssc.201900725 31132230

[B13] Gao C. Zhou J. Zhang Q. Cui P. Zhang Q. Wei W. (2023). Influence of LiBF4 sintering aid on the grain boundary and conductivity of LAGP electrolyte. Funct. Mater. Lett. 16, 2350001. 10.1142/s1793604723500017

[B14] Gao A. Lai H. Chen D. Huang W. Cheng X. Chen S. (2026). Operando XPS-guided interphase engineering of *in-situ* formed LiF-rich interphase to enable stable cycling of solid-state lithium batteries beyond 2500 hours. Energy Storage Mater. 88, 105066. 10.1016/j.ensm.2026.105066

[B15] Glynos E. Pantazidis C. Sakellariou G. (2020). Designing all-polymer nanostructured solid electrolytes: advances and prospects. ACS Omega 5, 2531–2540. 10.1021/acsomega.9b04098 32095677 PMC7033665

[B16] He Y. Zhu S. Gao S. Liu X. Li Z. Liang Y. (2026). Fluorinated polymer electrolyte via dual-salt coupling for solid-state lithium metal batteries. J. Mater. Chem. A 14 (20), 12256–12265. 10.1039/d5ta09340f

[B17] Hodge I. M. (1994). Enthalpy relaxation and recovery in amorphous materials. J. Non-Crystalline Solids 169, 211–266. 10.1016/0022-3093(94)90321-2

[B18] Hu L.-H. Wu F.-Y. Lin C.-T. Khlobystov A. N. Li L.-J. (2013). Graphene-modified LiFePO4 cathode for lithium ion battery beyond theoretical capacity. Nat. Commun. 4, 1687. 10.1038/ncomms2705 23575691

[B19] HuangJ. WangS. ChenJ. ChenC. LizundiaE. (2025). Environmental sustainability of natural biopolymer‐based electrolytes for lithium ion battery applications. Adv. Mater. 37, 2416733. 10.1002/adma.202416733 PMC1213884739757715

[B20] Jiang Y. Yan X. Ma Z. Mei P. Xiao W. You Q. (2018). Development of the PEO based solid polymer electrolytes for all-solid state lithium ion batteries. Polymers 10, 1237. 10.3390/polym10111237 30961162 PMC6401925

[B21] Jin M. Xu D. Su Z. Liu R. Xiang M. Cheng Y. (2025). Hydridoborate‐based solid electrolytes for all‐solid‐state batteries. Adv. Mater. 38, e07809. 10.1002/adma.202507809 40916154 PMC13549288

[B22] Lee J. J. (2018). Fabrication and characterizations of LAGP/PEO composite electrolytes for all solid-state lithium-ion batteries, Master's thesis. Wright State University.

[B23] Li S. Wang S. Du G. Liang J. Tong Z. Cui Y. (2025). Ultrathin inorganic-organic solid-state electrolyte reinforced by a pre-fiberized LAGP continuous skeleton. Sci. China Mater. 68, 199–206. 10.1007/s40843-024-3104-2

[B24] LiangH. WangL. WangA. SongY. WuY. YangY. (2023). Tailoring practically accessible polymer/inorganic composite electrolytes for all-solid-state lithium metal batteries: a review. Nano-Micro Lett. 15, 42. 10.1007/s40820-022-00996-1 PMC988959936719552

[B25] Liu W. Liu N. Sun J. Hsu P.-C. Li Y. Lee H.-W. (2015). Ionic conductivity enhancement of polymer electrolytes with ceramic nanowire fillers. Nano Lett. 15, 2740–2745. 10.1021/acs.nanolett.5b00600 25782069

[B26] Liu W. Lee S. W. Lin D. Shi F. Wang S. Sendek A. D. (2017). Enhancing ionic conductivity in composite polymer electrolytes with well-aligned ceramic nanowires. Nat. Energy 2, 17035. 10.1038/nenergy.2017.35

[B27] Ma T. Yu X. Cheng X. Li H. Zhu W. Qiu X. (2017). Confined solid electrolyte interphase growth space with solid polymer electrolyte in hollow structured silicon anode for Li-ion batteries. ACS Appl. Mater. and Interfaces 9, 13247–13254. 10.1021/acsami.7b03046 28374994

[B28] Perdew J. P. Burke K. Ernzerhof M. (1996). Generalized gradient approximation made simple. Phys. Rev. Lett. 77, 3865–3868. 10.1103/physrevlett.77.3865 10062328

[B29] Shin S.-R. Lee D.-S. (2024). Thermally healable polyurethane elastomers based on biomass polyester polyol from isosorbide and dimer fatty acid. Polymers 16, 3571. 10.3390/polym16243571 39771423 PMC11679297

[B30] ShinH. J. ParkS. W. ParkS. KimD. W. (2023). Polymer electrolyte/sulfur double‐shelled anisotropic reduced graphene oxide lamellar scaffold enables stable and high‐loading cathode for quasi‐solid‐state lithium‐sulfur batteries. Adv. Sci. 10, 2205424. 10.1002/advs.202205424 PMC995129736575365

[B31] Wang C. Liang J. Luo J. Liu J. Li X. Zhao F. (2021). A universal wet-chemistry synthesis of solid-state halide electrolytes for all-solid-state lithium-metal batteries. Sci. Adv. 7, eabh1896. 10.1126/sciadv.abh1896 34516879 PMC8442915

[B32] Wang H. Wang H. Shu K. Chen C. Liu Q. Cao R. (2026). Ba, Nb co‐doped LLZO reinforced PEO composite solid electrolyte for high‐performance solid‐state lithium batteries. J. Appl. Polym. Sci. 143, e70571. 10.1002/app.70571

[B33] Wang Y. He K. Yang J. Yang S. Lin T. Chen A. (2026). Designing an intergrowth‐structure Li ion‐sieve membrane for long‐term stable lithium extraction. Angew. Chem. 138, e25608. 10.1002/anie.202525608 41834445

[B35] Yu Z. Ren Y. Gao B. Wei M. Chen X. Qin H. (2026). Bioinspired adaptive coatings with quadruple-stimuli-responsive self-renewing and antibacterial micro/nano-textures for marine antifouling. Prog. Org. Coatings 211, 109791. 10.1016/j.porgcoat.2025.109791

[B36] Zhang S. Li Z. Guo Y. Cai L. Manikandan P. Zhao K. (2020). Room-temperature, high-voltage solid-state lithium battery with composite solid polymer electrolyte with *in-situ* thermal safety study. Chem. Eng. J. 400, 125996. 10.1016/j.cej.2020.125996

[B37] ZhangQ. HuangQ. HaoS. M. DengS. HeQ. LinZ. (2022). Polymers in lithium–sulfur batteries. Adv. Sci. 9, 2103798. 10.1002/advs.202103798 PMC880558634741443

[B38] Zhang N. Ren G. Li L. Wang Z. Yu P. Li X. (2024). Dynamical evolution of CO2 and H2O on garnet electrolyte elucidated by ambient pressure X-ray spectroscopies. Nat. Commun. 15, 2777. 10.1038/s41467-024-47071-4 38555416 PMC10981710

[B39] Zhao S. Zhang Y. Pham H. Carrillo J.-M. Y. Sumpter B. G. Nanda J. (2020). Improved single-ion conductivity of polymer electrolyte via accelerated segmental dynamics. ACS Appl. Energy Mater. 3, 12540–12548. 10.1021/acsaem.0c02079

