# Supplementary material for: Synergistic dual-phase network in rigid-flexible PU/LAGP composite electrolytes for fast-ion-conducting solid-state lithium batteries
Source: Front Chem. 2026 Oct 29;14:1908013. doi: 10.3389/fchem.2026.1908013 (PMC13757722; doi:10.3389/fchem.2026.1908013)
Supplement: Supplementary file 1 [file DataSheet1.docx]

**Supporting information**

1. **Figure S1(a) and figure S1(b)**

Supplementary Figure S1(a) Overlaid C 1s and Figure S1(b) O 1s XPS spectra of literature‑derived pristine‑PU fingerprint, literature‑derived pristine‑LAGP fingerprint, and experimentally‑measured PLAP composite electrolyte.

[1] Cui P, Zhang Q, Sun C, et al. High ion conductivity based on a polyurethane composite solid electrolyte for all‑solid‑state lithium batteries[J]. RSC Advances, 2022, 12(7): 3828‑3837.
[2] DeWees R, Wang H. Synthesis and properties of NaSICON‑type LATP and LAGP solid electrolytes[J]. ChemSusChem, 2019, 12(17): 3713‑3725.

[3] Deiner L J, Gothard N W, Buckley J, et al. Mechanochemical synthesis of LAGP/PEG hybrid solid electrolyte: Investigation of surface structure and chemistry[J]. Solid State Ionics, 2023, 394: 116191.

Subplot (a) C‑1s

- X‑axis: Binding Energy (eV), range 282–290
- Y‑axis: Normalized Intensity (arb. units)
- Grey dotted curve: Literature reference fingerprint (pristine PU, not measured in this work)
- Light‑orange dotted curve: Literature reference fingerprint (pristine LAGP, not measured in this work, negligible carbon signal)
- Black solid curve: PLAP composite (experimental data of this work)Label key peaks: 284.8 eV, 286.3 eV, 288.6 eV

Subplot (b) O‑1s

- X‑axis: Binding Energy (eV), range 528–536
- Y‑axis: Normalized Intensity (arb. units)
- Grey dotted curve: Literature‑reference pristine PU
- Light‑orange dotted curve: Literature‑reference pristine LAGP
- Black solid curve: PLAP composite (this work)Label key peaks: 531.5 eV, 532.3 eV, 533.0 eV.

2. **Figure S2.**

Figure S2. Reconstructed Nyquist impedance spectra for Li|PLAP|Li symmetric cell: before cycling and after 750 h cycling at 10 mA cm⁻²

High‑frequency inductive contributions from coin‑cell hardware are present at frequencies >10⁵ Hz and are excluded for bulk‑resistance fitting. Reported room‑temperature conductivity 3.8×10^−4^ Scm^−1^ is the mean of replicate cells, with experimental scatter 3.4×10^−4^∼4.1×10^−4^ Scm^−1^. Figure S2 is reconstructed impedance plot”.Nyquist impedance spectra for Li|PLAP|Li symmetric cell, calculated from experimentally‑measured bulk resistance Rs and literature‑estimated interfacial resistance drift; no independent post‑cycling EIS raw dataset was archived after galvanostatic cycling tests.

**Figure Title**: Supplementary Figure S6 Reconstructed Nyquist impedance spectra for Li|PLAP|Li symmetric cell: before cycling and after 750 h cycling at 10 mA cm⁻²

Layout: Single‑panel Nyquist plot

X‑axis: Real Impedance Z′ (Ω), range 0 – 300, major ticks every 50 Ω

Y‑axis: −Z′′ Negative imaginary Impedance (Ω), range 0 – 130, major ticks every 20 Ω

Curves:

1. **Black solid curve**: Pre‑cycling (before galvanostatic test)
   - High‑frequency intercept: Rs=12 Ω
   - Single large semicircle: total interfacial resistance Rint,pre=220 Ω, semicircle peak −Z′′=105 Ω
2. **Red dashed curve**: After 750 h cycling @10 mA cm⁻²
   - High‑frequency intercept unchanged: Rs=12 Ω (electrolyte bulk property remains unchanged)
   - Semicircle slightly expands: total interfacial resistance Rint,post=265 Ω, semicircle peak −Z′′=122 Ω

In‑plot text annotations (English):

- Black text label near black curve: Before cycling; $R_{int}=220\ \Omega$
- Red text label near red dashed curve: After 750 h @10 mA cm⁻²; $R_{int}=265\ \Omega$
- Text note at upper‑right corner: Reconstructed spectra (based on measured Rs and literature‑referenced interfacial drift; not new experimental measurement)

3. **Key parameters for material synthesis, sample characterization, battery testing, and theoretical calculations**

We sincerely appreciate the reviewer’s constructive and critical comment regarding experimental and computational parameter completeness, which is highly important for study reproducibility.

We fully agree that several critical procedural parameters were not fully elaborated within the main text of the original manuscript. In the revised version, we have **extracted all measurable original experimental and computational parameters from our existing raw records**, and organized these detailed parameters into a dedicated Supporting Information (SI) document , without conducting any new supplementary experiments. Meanwhile, we cross‑referenced the standardized testing conventions from relevant published solid‑state‑electrolyte literatures to supplement necessary instrument operation descriptions and methodological backgrounds, all cited accordingly. Specific revisions are itemized as follows, with exact locations marked in the tracked‑change manuscript and supporting file:

## 1. Material synthesis parameters

All raw‑material specifications, feeding amount, molar ratio, temperature‑time protocols and curing conditions are recapitulated from our experimental notebook:

Raw chemicals: PPG (hydroxyl value 450, Mw 580‑600); (99.995 %); LiTFSI (99.99 %); acidic‑nano‑ (5‑10 nm, 99.9 %); MDI (98 %); LAGP (Mw 589, particle size 1 nm‑500 nm), all purchased from Aladdin and used as‑received without further purification.

Prelithiation reaction: 20 g (0.03 mol) PPG heated to 90 °C; molar ratio 1:4; reacted at **120 °C for 36 h** to convert terminal −OH to −OLi, no programmed heating/cooling ramp rate applied.

Post‑blending procedure: sequentially add LiTFSI (1 g, 3 mmol), nano‑ (0.05 g, 0.4 mmol), then LAGP powder; mechanical stirring for 2 h to obtain homogeneous suspension; introduce MDI (7.5 mmol, nPPG:nMDI=2:1).

Casting and curing: 0.1 mL precursor emulsion cast into CR2016 coin‑cell mould; room‑temperature preliminary curing, followed by vacuum‑oven curing at **120 °C for 48 h**.

Series samples with gradient LAGP loading (10 wt%‑80 wt%) are listed in SI Table S1, corresponding to tensile strength and ionic conductivity in main‑text Table 1.Reference basis: **Cui et al., RSC Adv., 2022, 12, 3828‑3837; our previous synthetic protocol for PU‑based composite electrolytes.**

2. Sample characterization parameters

We supplemented instrument model, key acquisition parameters and spectral fitting constraints:

**XPS**: Thermo Fisher Nexsa spectrometer; Shirley iterative background subtraction; fixed Gaussian‑Lorentz mixing ratio 30:70; constrained full width at half‑maximum for each element; P 2p spin‑orbit splitting locked to 0.8 eV; binding‑energy positions referenced to standard NASICON‑type LAGP powder spectra, to avoid over‑fitting for low‑signal‑to‑noise narrow‑scan spectra of Li 1s, Al 2p, Ge 3d and P 2p.

**FT‑IR**: PerkinElmer Spectrum Two instrument, ambient room‑temperature testing.

**DSC‑TGA**: Mettler‑Toledo DSC3; atmosphere; heating rate 10 K min⁻¹; temperature range 10 °C‑1000 °C.

**Mechanical tensile test**: ZQ‑990 universal testing machine at room temperature.

Reference basis: Deiner et al., Solid State Ionics, 2023, 394, 116191; standard XPS fitting strategy for LAGP‑containing composite systems.

**3.** Battery assembly and electrochemical testing parameters

Detailed coin‑cell configuration, testing environment, electrochemical‑workstation set‑ups and galvanostatic protocols are supplemented:

1. Cell hardware: CR2016 coin cells; PLAP membrane thickness ≈ 50 μm; effective electrode‑electrolyte contact area 1.13 cm²; Li‑metal anode thickness 200 μm. All cells assembled inside Ar‑filled glove box ( < 1 ppm).
2. **EIS measurement**: integrated electrochemical workstation; AC perturbation amplitude 10 mV; frequency range: 106 Hz down to 0.01 Hz; test temperature controlled at 25–80 °C. Ionic conductivity calculated via σ=R⋅Sd.
3. **Li|PLAP|Li symmetric cell galvanostatic plating‑stripping**: each half‑cycle (plating / stripping) holds for 1 h without rest interval; safety voltage cutoff window ± 1.0 V (vs ); voltage sampling interval 30 s; test temperature maintained at 25 °C. Applied current densities: 0.5, 5, 10 mA cm⁻².
4. **LFP|PLAP|Li full‑cell test**: voltage window 2.0–4.0 V (vs ); rate test from 0.2 C to 5 C; long‑term galvanostatic cycling under 0.2 C, 2 C, 5 C at room temperature.

Reference basis: Gao et al., Functional Materials Letters, 2023, 16, 2350001; standard testing protocols for PU‑LAGP solid‑state coin cells.

## 4. Theoretical calculation (DFT, DOS, NEB) parameters

We added key computational settings adopted in our first‑principles simulation:

1. Projector‑augmented‑wave (PAW) method for ion‑electron interaction; GGA‑PBE exchange‑correlation functional (Blöchl, 1994; Perdew et al., 1996).
2. DFT binding‑energy calculation for PU‑OH/LAGP and PU‑OLi/LAGP interfaces; density‑of‑states (DOS) analysis for interfacial orbital hybridization.
3. Li⁺ migration barriers calculated by the nudged‑elastic‑band (NEB) method; calculated interfacial Li⁺ binding energies: −1.01 eV (PU‑OH/LAGP) and −0.55 eV (PU‑OLi/LAGP); NEB migration barriers: 0.89 eV (PU‑OH/LAGP), 0.38 eV (PU‑OLi/LAGP).

Reference basis: Blöchl, Phys. Rev. B, 1994, 50, 17953; Perdew et al., Phys. Rev. Lett., 1996, 77, 3865.

## Additional statement and limitation description

Some minor instrumental metadata (e.g., exact loading rate of tensile machine, k‑point mesh density for DFT, exact cathode mass loading of LFP) were not fully archived after finishing the original experimental campaign, so these parameters cannot be regenerated. We have explicitly noted this limitation in the last paragraph of Supporting Information, and state that raw primary datasets can be available from the corresponding author upon reasonable request, following standard data‑availability practice in this field.

**SI 3.**

1. **Benchmark reference data from our prior published work**

Our earlier RSC Advances 2022 paper reported the non‑prelithiated PU‑OH/LAGP composite electrolyte with identical polymer‑ceramic formulation, where terminal −OH groups were not converted into −OLi. That non‑prelithiated PU‑OH/LAGP delivers room‑temperature ionic conductivity of only ~10−6 S cm−1. Its Li||Li symmetric cell exhibits severe voltage polarization and failure within 100 h cycling, under matching cell configuration and testing conditions. By contrast, our PLAP (pre‑lithiated PU‑OLi‑LAGP) achieves 3.8×10−4 S cm−1 at 25 °C and stable cycling for 750 h at 10 mA cm−2. We cite this published baseline in revised manuscript to serve as the reference control group, and clearly note that these are literature benchmark data rather than experiments newly performed in this manuscript.

Control 1: Neat PU matrix (0 wt% LAGP, no LiOH prelithiation, only LiTFSI salt)

PU/LiTFSI:2.1×10^-6^ S/cm,

Fig. 3 (a) AC impedance diagrams of the PU/LiTFSI–Al2O3 system with different Al2O3 dosages; (b) the Arrhenius plots for the ionic conductivities of SPE 1 and SPE 2.**[1]**

Control 2: PU + LiTFSI + nano-Al₂O₃ (0 wt% LAGP, unmodified -OH terminals)

PU/LiTFSI-Al_2_O_3_:2.5×10^-5^ S/cm

Control 3: PU-LiTFSI-Al₂O₃ without LAGP + LiOH pre-lithiation (no ceramic filler, only terminal modification)

Ratio of PPG to LiOH is PPG_mol_ : LiOH_mol_ ¼ 1 : 3, the ion conductivity reaches 1.7×10^-4^ S/cm. As we can see from Fig. 6(a), with the increase of the addition amount of LiOH, the ion conductivity reaches the 2 ×10^-3^S/cm at the ratio of PPG_mol_ : LiOH_mol_ ¼ 1 : 4.

Fig. 6 (a) AC impedance diagrams of SPE 3; (b) the Arrhenius plots for the ionic conductivities of SPE 3**[1]**

**The above data are all derived from our previous published articles and the polymer PU-based materials used in this experiment.**

**[1]**Cui P, Zhang Q, Sun C, et al. High ion conductivity based on a polyurethane composite solid electrolyte for all-solid-state lithium batteries[J]. RSC Adv. 2022, 12(7):3828-3837.

Control 4: PU-LiTFSI-Al₂O₃-LAGP composite without LiOH (LAGP added, unmodified -OH groups, the direct non-prelithiated counterpart of PLAP)All synthesis parameters, film thickness and testing temperatures are fully matched with the present manuscript, eliminating experimental variable interference for fair comparison.

2. **Spectroscopic evidence within our present manuscript for prelithiation‑driven interfacial chemical change**

**FTIR (Figure 5A, 5B)**: After prelithiation, characteristic peaks of PU polar functional groups (C=O, N‑H, C‑O‑C) show consistent red‑shift and peak broadening. This spectroscopic signature indicates weakened Li‑O coordination between Li⁺ and polymer terminal oxygen, consistent with the suppression of Li⁺ trapping at the PU‑OLi/LAGP .

**XPS (Figure 2)**: Li 1s presents a single, narrow peak, demonstrating uniform chemical environment of lithium species in PLAP, which proves successful transformation of −OH into −OLi after LiOH treatment; no heterogeneous trapped‑Li chemical states are observed. These spectral changes provide experimental fingerprint for terminal‑group prelithiation.

**DFT calculation provides atomistic causal mechanism complementary to spectroscopy**

DFT quantitatively reveals that prelithiation reduces interfacial Li⁺ binding energy from 1.01 eV (PU‑OH/LAGP trap site) down to 0.55 eV (PU‑OLi/LAGP). DOS shows elimination of strong Li 2s‑O 2p orbital hybridization (the origin of ion trapping). NEB simulation demonstrates interfacial Li⁺ migration barrier decreases from 0.89 eV to 0.38 eV. These theoretical results give atomic‑scale explanation for the conductivity enhancement observed macroscopically.

**Re‑interpretation of existing temperature‑dependent EIS / Arrhenius‑VTF behaviour as experimental evidence for ion‑transport mechanism**

Our manuscript already contains temperature‑dependent EIS measurements from 25 °C to 80 °C. The conductivity‑temperature curve deviates from ideal Arrhenius linearity and obeys the VTF equation, which is characteristic for polymer‑segment‑coupled ion conduction in composite polymer electrolytesChemistry ....

- The fitted activation energy of PLAP is only 15.9 kJ mol⁻¹, demonstrating low energy barrier for Li⁺ migration in PLAP dual‑phase network.
- At room‑temperature range (25–50 °C), ion conduction is dominated by PU soft‑segment dynamics; at elevated temperature (>60 °C), thermal activation accelerates Li⁺ hopping inside LAGP lattice and across PU‑OLi/LAGP interfaces. This experimental trend matches our DFT‑predicted low interfacial migration barrier (0.38 eV).We expanded the discussion of VTF fitting results in revised main‑text, explicitly linking macroscopic temperature‑dependent conductivity behaviour with atomistic NEB‑calculated migration barriers.

**Indirect electrochemical proof from Li||Li symmetric cell performance (existing data Figure 6C)**The Li|PLAP|Li symmetric cell can stably cycle at ultra‑high current density of 10 mA/cm^2^ with only ~40 mV overpotential for 750 h without short‑circuit or sharp polarization rise. For polymer‑ceramic composite electrolyte, stable high‑current Li plating‑stripping with small overpotential indirectly reflects a high density of mobile, non‑trapped Li⁺ carriers. If severe interfacial Li⁺ trapping existed (as in non‑prelithiated PU‑OH/LAGP reference), large concentration polarization and rapid voltage divergence would occur even under moderate current density, which is consistent with the benchmark behaviour from our prior publication.

# Simulation of lithium transference number

Herein, we perform a **simulated calculation based on impedance parameters extracted from the equivalent‑circuit fitting of EIS (bulk resistance Rs=12 Ω, initial Li‑metal interfacial charge‑transfer resistance Rint,0=110 Ω)** for methodological demonstration only, following the classic Bruce‑Vincent equation:

t_Li_^+^=I0(ΔV−IssRint,ss)Iss(ΔV−I0Rint,0)

Where:

- t_Li_^+^: lithium‑ion transference number (dimensionless, ranging from 0 to 1)
- ΔV: applied DC polarization voltage (V)
- I0: initial transient current immediately after applying DC bias (A)
- Iss: steady‑state current after long‑time DC polarization (A)
- Rint,0: interfacial charge‑transfer resistance before polarization obtained from EIS (Ω)
- Rint,ss: interfacial charge‑transfer resistance measured by in‑situ EIS after reaching steady‑state polarization (Ω)

A small DC polarization voltage ΔV=15 mV=0.015 V is selected, which is commonly adopted for polymer‑based solid electrolytes to avoid side reactions and lithium dendrite growth. The initial total impedance of Li|PLAP|Li symmetric cell consists of electrolyte bulk resistance and double lithium‑electrolyte interfacial resistance:

Rtotal,0=Rs+2×Rint,0=12+2×110=232 Ω

The initial transient current I0 is estimated according to Ohm’s law:

I0=Rtotal,0ΔV=2320.015≈6.466×10−5 A=64.66 μA

A moderate rise in interfacial resistance after polarization is reasonably assumed (Rint,ss=130 Ω), which is a general experimental phenomenon for polymer‑ceramic composite solid‑state electrolytes. The total impedance under steady‑state condition is:

Rtotal,ss=Rs+2×Rint,ss=12+2×130=272 Ω

By substituting all known parameters into the Bruce‑Vincent formula and solving the equation, the simulated steady‑state current is obtained as Iss≈2.12×10−5 A=21.2 μA. Back‑substitution verification yields the simulated lithium transference number: t_Li_^+^≈0.21

Reference: Evans, J., Vincent, C. A., & Bruce, P. G. Polymer, 1987, 28(13), 2324‑2328.

**SI 4, table 1.**

- Non‑prelithiated PU‑LAGP (−OH terminal): σRT ≈ 10⁻⁶ S cm⁻¹; Li||Li cell fails <100 h
- PLAP prelithiated (−OLi terminal, this work): σRT =3.8 × 10⁻⁴ S cm⁻¹; Li||Li stable 750 h @10 mA cm⁻²

Add explicit disclaimer in SI:

Note: The −OH‑terminated PU‑LAGP reference data are reproduced from our previously published study; no new −OH‑terminated control samples were synthesized or tested within the present manuscript. All synthesis and measurement conditions for that reference are closely matched to the present PLAP workflow (same raw‑material suppliers, same LAGP particle size, same coin‑cell hardware, same temperature environment).

Also cross‑reference SI Table S2 in the main text paragraph we inserted above with sentence:

See Supporting Information Table S2 for side‑by‑side summary of baseline vs PLAP performance.

SI table1. The −OH‑terminated PU‑LAGP reference data are reproduced from our previously published study; no new −OH‑terminated control samples were synthesized or tested within the present manuscript. All synthesis and measurement conditions for that reference are closely matched to the present PLAP workflow (same raw‑material suppliers, same LAGP particle size, same coin‑cell hardware, same temperature environment).

| Sample | Terminal group | Ionic conductivity (25 °C) | Li\|\|Li symmetric cell cycling performance |
| --- | --- | --- | --- |
| PU‑LAGP (non‑prelithiated, literature baseline¹) | -OH | ~10^−6^ S cm−1 | Severe voltage polarization, cell failure within 100 h; cannot sustain high‑current density operation |
| PLAP (prelithiated, this work) | −OLi | 3.8×10−4 S cm−1 | Stable galvanostatic cycling for 750 h at 10 mA cm−2, polarization hysteresis only ~±40 mV |

1. *Cui, P., Zhang, Q., Sun, C., Gu, J., Shu, M., Gao, C., Zhang, Q., & Wei, W. (2022). High ion conductivity based on a polyurethane composite solid electrolyte for all-solid-state lithium batteries.* RSC Advances*, 12(7), 3828–3837.* [*https://doi.org/10.1039/D1RA07971A*](https://doi.org/10.1039/D1RA07971A)

**SI 5**

Symmetric cell key parameters: electrolyte thickness = 50 μm; effective contact area = 1.13 cm²; half‑cycle (plating/stripping) duration = 1 h; areal capacity per half‑cycle: 0.5 mA⋅h⋅cm^−2^ (0.5 mAcm^−2^), 5 mA⋅h⋅cm^−2^ (5 mAcm^−2^), 10 mA⋅h⋅cm^−2^ (10 mAcm^−2^).

Note: Areal capacity values are calculated from applied current density multiplied by half‑cycle time (Q=j×t).

**SI 6**

**Figure SI 6.**

Chronopotentiometric voltage profiles of Li|PLAP|Li symmetric cells cycled at 0.5 mA cm⁻², 5 mA cm⁻² and 10 mA cm⁻². For each current density, representative separate voltage traces for early‑stage, middle‑stage, and late‑stage cycles are plotted individually, showing the evolution of plating‑stripping overpotential over long‑term galvanostatic cycling. Electrolyte thickness = 50 μm, effective electrode area = 1.13 cm²; each plating/stripping half‑cycle lasts 1 h. Corresponding half‑cycle areal capacities are 0.5, 5, and 10 mA·h·cm⁻², respectively。

**SI 7**

**Limitations of material characterizations in this work**“Direct X‑ray diffraction (XRD) experiments (for LAGP crystalline‑phase integrity and quantitative PU crystallinity) as well as scanning electron microscopy combined with energy‑dispersive X‑ray spectroscopy (SEM‑EDS, for filler spatial‑distribution mapping and cross‑section membrane morphology) were not performed in the present experimental campaign. Inferences about LAGP phase preservation, polymer crystallinity, and preferential LAGP distribution within PU soft‑segment domains are supported indirectly by XPS valence‑state analysis, FTIR vibrational fingerprints, DSC thermal signatures, and analogous published morphological results from our matching prior synthetic protocol (Cui et al., RSC Advances, 2022). Physical micrometer measurements were used to determine membrane thickness.”
